# The conserved Spd-2/CEP192 domain adopts a unique protein fold to promote centrosome scaffold assembly

**DOI:** 10.1126/sciadv.adr5744

**Published:** 2025-03-19

**Authors:** Liuyi Hu, Alan Wainman, Antonina Andreeva, Muladili Apizi, Ines Alvarez-Rodrigo, Siu-Shing Wong, Saroj Saurya, Devon Sheppard, Matthew Cottee, Steven Johnson, Susan M. Lea, Jordan W. Raff, Mark van Breugel, Zhe Feng

**Affiliations:** ^1^ State Key Laboratory of Genetic Engineering, School of Life Sciences, Fudan University, Shanghai, China.; ^2^ Sir William Dunn School of Pathology, University of Oxford, Oxford OX1 3RE, UK.; ^3^ Medical Research Council—Laboratory of Molecular Biology, Francis Crick Avenue, Cambridge CB2 0QH, UK.; ^4^ Francis Crick Institute, London NW1 1AT, UK.; ^5^ Center for Structural Biology, CC R, NCI, Frederick, MD 21702-1201, USA.

## Abstract

Centrosomes form when centrioles assemble pericentriolar material (PCM) around themselves. Spd-2/CEP192 proteins, defined by a conserved “Spd-2 domain” (SP2D) comprising two closely spaced AspM-Spd-2-Hydin (ASH) domains, play a critical role in centrosome assembly. Here, we show that the SP2D does not target *Drosophila* Spd-2 to centrosomes but rather promotes PCM scaffold assembly. Crystal structures of the human and honeybee SP2D reveal an unusual “extended cradle” structure mediated by a conserved interaction interface between the two ASH domains. Mutations predicted to perturb this interface, including a human mutation associated with male infertility and Mosaic Variegated Aneuploidy, disrupt PCM scaffold assembly in flies. The SP2D is monomeric in solution, but the *Drosophila* SP2D can form higher-order oligomers upon phosphorylation by PLK1 (Polo-like kinase 1). Crystal-packing interactions and AlphaFold predictions suggest how SP2Ds might self-assemble, and mutations associated with one such potential dimerization interface markedly perturb SP2D oligomerization in vitro and PCM scaffold assembly in vivo.

## Teaser

A conserved structural domain within CEP192/Spd-2 protein promotes centrosome scaffold assembly during centrosome maturation.

## INTRODUCTION

Centrosomes are important cellular organizers in many species, and they form when a mother centriole recruits pericentriolar material (PCM) around itself (*1*, *2*). The PCM contains several hundred proteins (*3*), allowing centrosomes to function as major microtubule (MT)–organizing centers and important coordination centers within many cell types (*4*, *5*). Centrosome dysfunction has been linked to a plethora of human diseases and developmental disorders (*6*–*8*).

During interphase, the mother centriole recruits a relatively small amount of PCM that is tightly organized around the mother centriole (*9*–*12*). As cells prepare to enter mitosis, the PCM expands markedly around the mother centriole in a process termed centrosome maturation (*1*, *13*). Electron microscopy studies suggest that centrioles organize an extensive “scaffold” structure during mitosis that expands outward around the mother centriole and that recruits many other PCM “clients” such as the γ-tubulin ring complex (*14*–*16*).

The conserved centriole/centrosome protein Spd-2/CEP192 (fly/human nomenclature) plays an important part in mitotic centrosome assembly in humans (*17*, *18*), flies (*19*–*21*), and worms (*22*, *23*) and also in the diverged centrosomes of *Dictyostelium*, which lack canonical centrioles (*24*). In flies and worms, the phosphorylation of Spd-2/SPD-2 at centrioles recruits Polo/Polo-like kinase 1 (PLK1), which then phosphorylates the large coiled-coil protein Cnn/SPD-5 to stimulate the assembly of a Cnn scaffold (*21*, *25*, *26*), and the internal molecular interactions that allow Cnn and SPD-5 to assemble into scaffolds are beginning to be elucidated (*27*–*31*). Mitotic centrosome maturation is abolished in the absence of this pathway in flies and worms, and the human homologs of Spd-2, Cnn, and Polo (CEP192, CDK5RAP2, and PLK1, respectively) play an important part in mitotic centrosome assembly (*17*, *18*, *32*–*38*). Intriguingly, experiments in flies show that Spd-2 can still organize a scaffold structure in the absence of Cnn; this scaffold is less robust than normal, but it can still recruit many PCM clients (*39*). Here, we investigate how Spd-2/CEP192 proteins contribute to PCM scaffold assembly.

Spd-2/CEP192 proteins are defined by the presence of an Spd-2 domain (hereafter SP2D) (*23*), which is composed of two closely spaced AspM-Spd-2-Hydin (ASH) domains (*40*). An evolutionary analysis reveals that most Spd-2/CEP192 proteins contain additional ASH domains located close to the SP2D domain (one in *Drosophila melanogaster* and six in *Homo sapiens*; see Results). ASH domains are present in several proteins associated with centrosomes, cilia, flagella, and the Golgi complex, but the functions of these domains remain largely unknown. Here, we show that, perhaps unexpectedly, the SP2D is not required to target Spd-2 to centrosomes in flies. Instead, *Dm*SP2D is essential for PCM scaffold assembly, a function that does not appear to require the additional ASH3 domain.

The crystal structures of the human and honeybee SP2D reveal that the two ASH domains form immunoglobulin G–like folds held together in an unusual “extended cradle” conformation. This unique structure appears to be the defining feature of the SP2D, and AlphaFold2 (AF2) predictions suggest that the SP2Ds of many other species, including *Drosophila*, adopt a similar fold. Mutations predicted to disrupt the extended cradle structure in the *Drosophila* SP2D perturb PCM scaffold assembly in vivo. Moreover, a conserved asparagine (Asn) located close to this interaction interface was recently shown to be mutated in humans, and the associated Asn1917Ser substitution is linked to male infertility and Mosaic Variegated Aneuploidy (MVA) syndrome (*41*). We show that the equivalent substitution in *Drosophila* Spd-2 mildly perturbs scaffold assembly in vivo, suggesting that mildly defective mitotic centrosome assembly may be the underlying cause of the human pathology. Last, we present evidence that hydrophobic packing interactions between different SP2Ds identified in the crystal structure and AF3 dimer predictions could play a part in allowing SP2Ds to assemble into higher-order structures.

## RESULTS

### Structure of Spd-2/CEP192 proteins

**Fig. 1.**
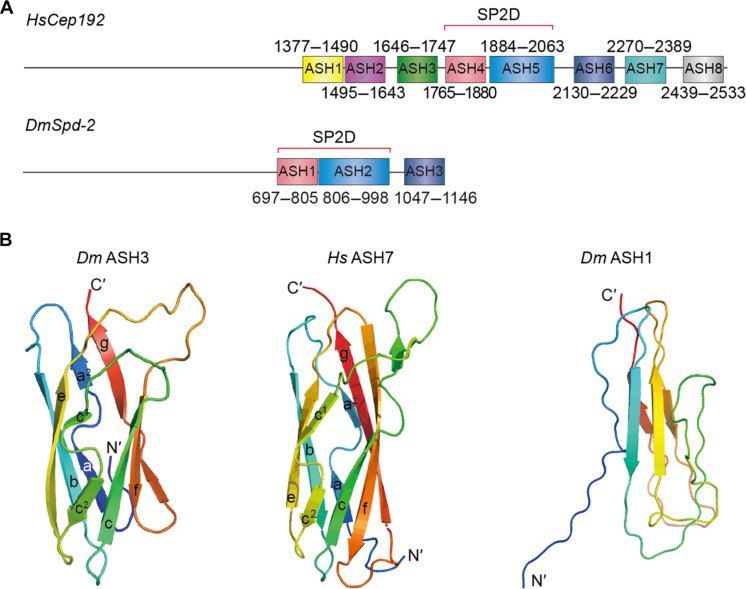
Domain organization of Spd-2/CEP192 proteins. (**A**) Schematics illustrate the domain organization of *H. sapiens* (*Hs*) CEP192 and *D. melanogaster* (*Dm*) Spd-2 proteins. (**B**) Ribbon representation of structures determined for *Dm*ASH3 and *Hs*ASH7 by crystallography and *Dm*ASH1 by NMR spectroscopy.

The N-terminal 696 amino acids of *Drosophila* Spd-2 and 1340 amino acids of human CEP192 were predicted to be largely unstructured by PONDR (*42*) and by AF2 (*43*) (Fig. 1A). AF2 predictions of *Drosophila* and human CEP192 suggested that their C-terminal regions—residues 697 to 1146 (*Drosophila*) and 1377 to 2533 (human)—have a predominantly β-secondary structure and contain a variable number of previously identified ASH domains (Fig. 1A and fig. S1A). To partially validate the AF2 structural models, we solved the high-resolution structures of individual ASH domains from *Dm*Spd-2 and *H. sapiens* CEP192 by crystallography (*Dm*ASH3 and *Hs*ASH7) to resolutions of 1.93 and 1.0 Å (Table 1), respectively, or by nuclear magnetic resonance (NMR) spectroscopy (*Dm*ASH1) (Table 2). All these proteins were predominantly monomeric in solution, and they adopted a similar immunoglobulin-like fold that is common to members of the PapD-like superfamily (Fig. 1B and fig. S1B). There was a lack of defined secondary structural elements in purified *Dm*ASH1, suggesting that this domain alone (i.e., when not in its normal context with *Dm*ASH2 in the SP2D) was intrinsically flexible, potentially explaining why we could not crystallize it despite its high degree of purity and homogeneity.

**Table 1.** Data collection and refinement statistics.

	*Drosophila* Spd-2 ASH3 domain	SeMet *A. dorsata* CEP192^946–1284^ (peak)	SeMet *H. sapiens* CEP192^2256–2402^ (remote)
Data collection			
Beamline	Diamond I04-1	Diamond I24	ESRF BM14
Space group	*I*4_1_22	*I*4_1_	*P*22_1_2_1_
Wavelength (Å)	0.92	0.979	0.729
Cell dimensions (Å)			
a, b, c (Å)	88.6, 88.6, 79.0	183.3, 183.3, 190.0	39.7, 60.1, 64.1
α, β, γ (°)	90, 90, 90	90, 90, 90	90, 90, 90
Resolution range (Å)^*^	62.64–1.93	49.48–3.50	23.03–1.00
Rmerge^*^^†^	0.081 (0.566)	0.121 (1.038)	0.071 (1.376)
Rpim^*^^‡^	0.038 (0.342)	0.056 (0.483)	0.019 (0.368)
Mean I/sI^*^	15.1 (2.4)	10.4 (1.9)	18.2 (2.3)
Completeness (%)^*^	99.1 (99.4)	100.0 (100.0)	98.8 (97.2)
Multiplicity^*^	5.6 (4.4)	5.7 (5.6)	14.8 (14.8)
Wilson <B> (Å2)	29.5	84.6	8.6
Refinement			
Resolution range (Å)	62.64–1.93 (2.12–1.93)	43.29–3.50 (3.59–3.50)	23.03–1.00 (1.03–1.00)
No. of reflections	12002 (2936)	39490 (2815)	78331 (5907)
Rwork/Rfree	0.177/0.212	0.288/0.313	0.136/0.152
Number of atoms			
Protein	786	16742	1111
Ligand/ion	27	N/A	1
Water	91	N/A	198
B-factors (Å2)	42.1	116.50	15.2
RMSD from ideal values			
Bond lengths (Å)	0.01	0.003	0.014
Bond angles (°)	1.05	0.746	1.827
Ramachandran plot			
Favored region (%)	96.9	95.62	98.52
Allowed (%)	3.1	4.38	0.74
Outliers (%)	0	0	0.74
Rotamer outliers (%)	0	0.05	1.14
C-beta outliers	0	0	1
PDB ID code	9C72	9FH8	6FVI

*Values in parentheses are for the highest resolution shell. A single crystal was used for the structure determination.

^†^$R_{\text{merge}}=\Sigma \left(I_{\mathrm{hl}}-<I_{h}>\right)/\Sigma \left(I_{\mathrm{hl}}\right)$, where $<I_{h}>$ is the mean intensity of unique reflection *h*, summed over all reflections for each observed intensity $I_{\mathrm{hl}}$.

^‡^$R_{\text{pim}}=\Sigma \sqrt[{2}]{1/\left(N-1\right)\;\left(I_{\mathrm{hl}}-<I_{h}>\right)/\Sigma \left(I_{\mathrm{hl}}\right)}$.

**Table 2.** NMR structure refinement statistics.

	*Drosophila* Spd-2 ASH1 domain
Total NOEs	1220
Intraresidue	380
Sequential	417
Medium-range <5	93
Long-range ≥5	330
Dihedral angle restraints	114
Ramachandran (procheck)	
Residues in most favored (%)	83.4
Residues in allowed (%)	16.6
Residues in generously allowed (%)	0.0
Residues in disallowed (%)	0.0
RMSD (Å)	
Backbone	1.4
Heavy atoms	1.8
Restraint violations	
Distance violations >0.5 Å	0
Angle violations	0
Deviations from ideal geometry	
Close contacts	0
RMS for bond angles (°)	0.2
RMS for bond lengths (Å)	0.001

We used the human CEP192 AF2 prediction model as a guide and constructed novel Pfam domain families (*44*) of individual ASH domains (fig. S2). The number of identified CEP192/Spd-2 ASH domains varied across species, with most of those analyzed containing eight ASH domains but others containing seven, six, three, or two (fig. S2B)—a conclusion supported by further selected AF2 predictions (fig. S1, C to F). The ASH1 and ASH2 domains in flies, and equivalent ASH4 and ASH5 domains in humans, collectively form the most conserved “Spd-2-domain” (SP2D) (fig. S1G) (*23*)—but even these ASH domains appear to have diverged in position and sequence in some species (fig. S2).

### Mapping potential functions for the different regions within *Dm*Spd-2

**Fig. 2.**
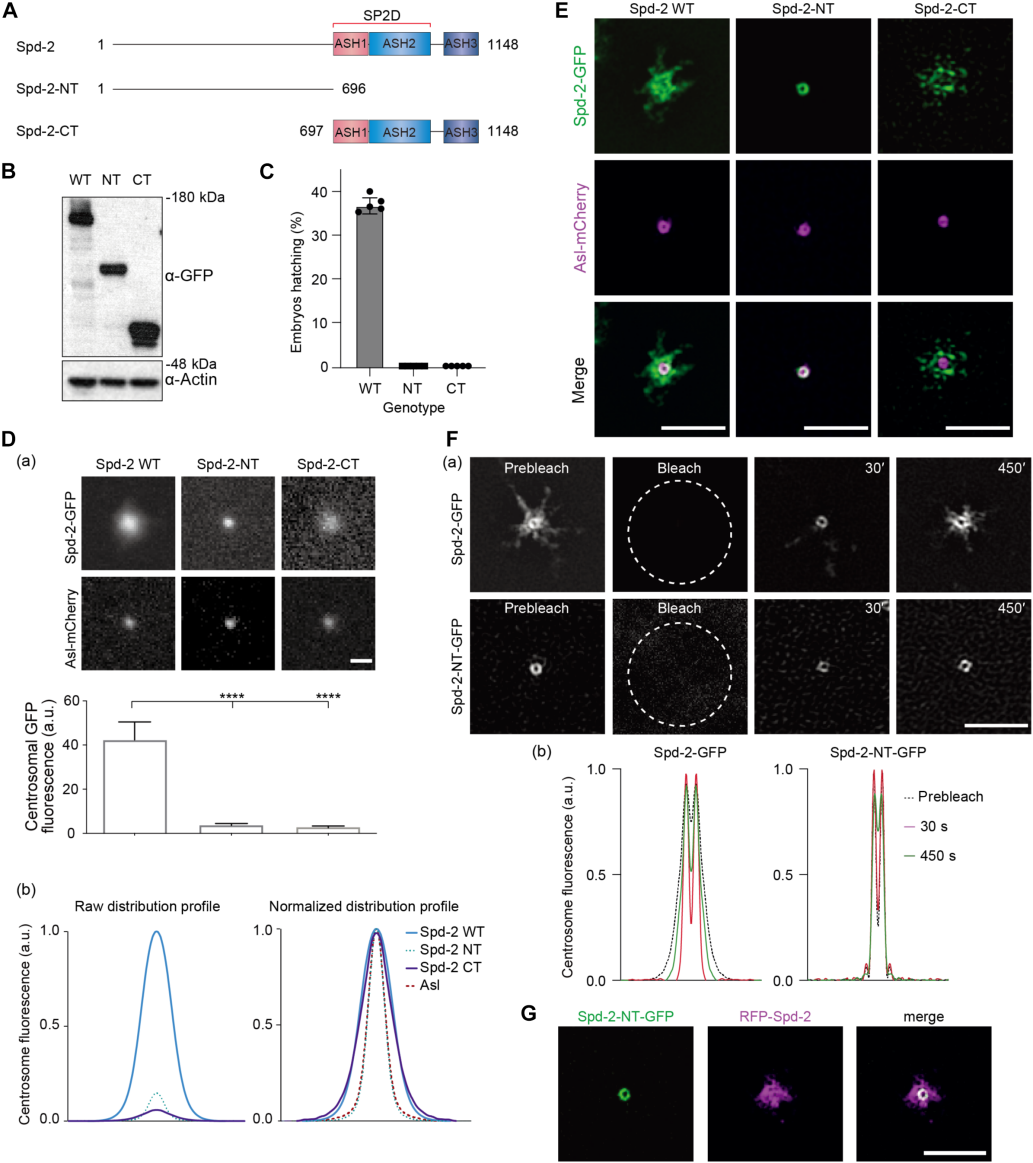
The N-terminal region of *Dm*Spd-2 is sufficient for centriole targeting, and the C-terminal region is required for PCM scaffold assembly. (**A**) Schematics illustrate the Spd-2 truncation mutants tested here. (**B**) Western blot shows the expression levels of GFP fusions to WT or the truncated versions of Spd-2 in early embryos; actin is shown as a loading control. NT, N-terminal; CT, C-terminal. (**C**) Graphs quantify the hatching percentage of embryos laid by *Spd-2^−/−^* females expressing a GFP fusion of either WT Spd-2 or the truncation mutants. *n* = 5 technical repeats; *n* ≥ 100 embryos per technical repeat. (**D**) (a) Confocal images illustrate, and the bar chart below quantifies, the centrosomal fluorescence intensity (means ± SD) of the WT and truncated Spd-2-GFP fusions in WT embryos expressing the centriole marker Asl-mCherry and injected with mRNA encoding each GFP fusion. Note that endogenous, unlabeled Spd-2 is also present in these embryos. Statistical significance was assessed using an unpaired *t* test in GraphPad Prism (****P < 0.0001). a.u., arbitrary units. (b) Graphs show the raw (left) or normalized (right) centrosomal fluorescence distribution profiles of the WT or truncated Spd-2-GFP fusions. (**E**) 3D-SIM images of centrosomes in living embryos expressing the mother centriole marker Asl-mCherry (magenta) injected with mRNAs encoding either Spd-2 WT-GFP or its truncated mutants (green). (**F**) (a) 3D-SIM images from a FRAP experiment show the dynamic behavior of WT Spd-2-GFP and Spd-2-NT-GFP at centrosomes. The time (seconds) after photobleaching is indicated. (b) Graphs show the normalized centrosomal fluorescence distribution profiles of the WT or truncated Spd-2-GFP fusions at successive time points after photobleaching. Note how the WT protein appears to spread outward, but Spd-2-NT-GFP remains tightly concentrated around the centriole. (**G**) 3D-SIM images of centrosomes in embryos coexpressing Spd-2-NT-GFP and full-length WT RFP-Spd-2. Scale bars in all images, 2 μm.

To gain insight into the potential function of the different regions within Spd-2/CEP192 proteins, we used *Drosophila* Spd-2 as a model. We generated transgenic lines expressing green fluorescent protein (GFP) fusions of either full-length wild-type (WT) Spd-2 (Spd-2-GFP) (*19*) or deleted forms containing only the N-terminal (Spd-2-NT-GFP) or C-terminal (Spd-2-CT-GFP) regions (Fig. 2A). Western blotting revealed that all three proteins were expressed in embryos at similar levels (Fig. 2B). Centrosomes are not essential for most of *Drosophila* development (*45*), but they are essential for the rapid rounds of nuclear division that occur in the early embryo (*46*, *47*). Therefore, *Spd-2* mutant flies are viable, but mutant females lay embryos that die in early development (*19*, *20*). Neither Spd-2-NT-GFP nor Spd-2-CT-GFP could rescue this early embryo lethality (Fig. 2C), demonstrating that both the N- and C-terminal regions are required for the Spd-2 function.

We used standard spinning disk confocal microscopy (Fig. 2D), super-resolution three-dimensionally structured illumination microscopy (3D-SIM) (Fig. 2E), and 3D-SIM combined with fluorescence recovery after photobleaching (FRAP) (Fig. 2F and movie S1) to assess the behavior of the WT and deleted forms of Spd-2-GFP protein. This revealed that, as shown previously (*39*), Spd-2-GFP was initially recruited to a tight ring around the mother centriole—marked in these experiments with Asl-mCherry (*1*)—and it then assembled into a larger structure (hereafter a “scaffold”) that fluxes outward around the mother centriole (Fig. 2, E and F, and movie S1) (*2*). Like WT Spd-2-GFP, Spd-2-NT-GFP was initially recruited to the mother centriole, but it did not detectably form a scaffold, and so, its total centrosomal levels were greatly reduced (Fig. 2, D to F). These experiments were performed in WT embryos that contain endogenous, unlabeled WT Spd-2, suggesting that Spd-2-NT-GFP could not incorporate into the WT PCM scaffold that was presumably being organized by the untagged WT Spd-2. This was confirmed in embryos coexpressing Spd-2-NT-GFP with RFP-Spd-2 (Fig. 2G). Thus, Spd-2-NT-GFP is recruited to centrioles, but it cannot form a scaffold, and it cannot incorporate into any scaffold formed by WT Spd-2.

The centrosomal recruitment of Spd-2-CT-GFP was also greatly reduced compared to WT Spd-2-GFP (Fig. 2D), but unlike Spd-2-NT-GFP, this protein was no longer detectable at centrioles, and instead, it exhibited a very low-level binding throughout the PCM (Fig. 2, D and E). This is consistent with previous data indicating that Spd-2 can only efficiently incorporate into the PCM if it has first been recruited to centrioles (*21*, *26*, *39*). Together, these data indicate that, in flies, the N-terminal region targets Spd-2 to the centriole, while the C-terminal region allows Spd-2 to assemble into a PCM scaffold.

### Biological role of the SP2D in PCM scaffold assembly

**Fig. 3.**
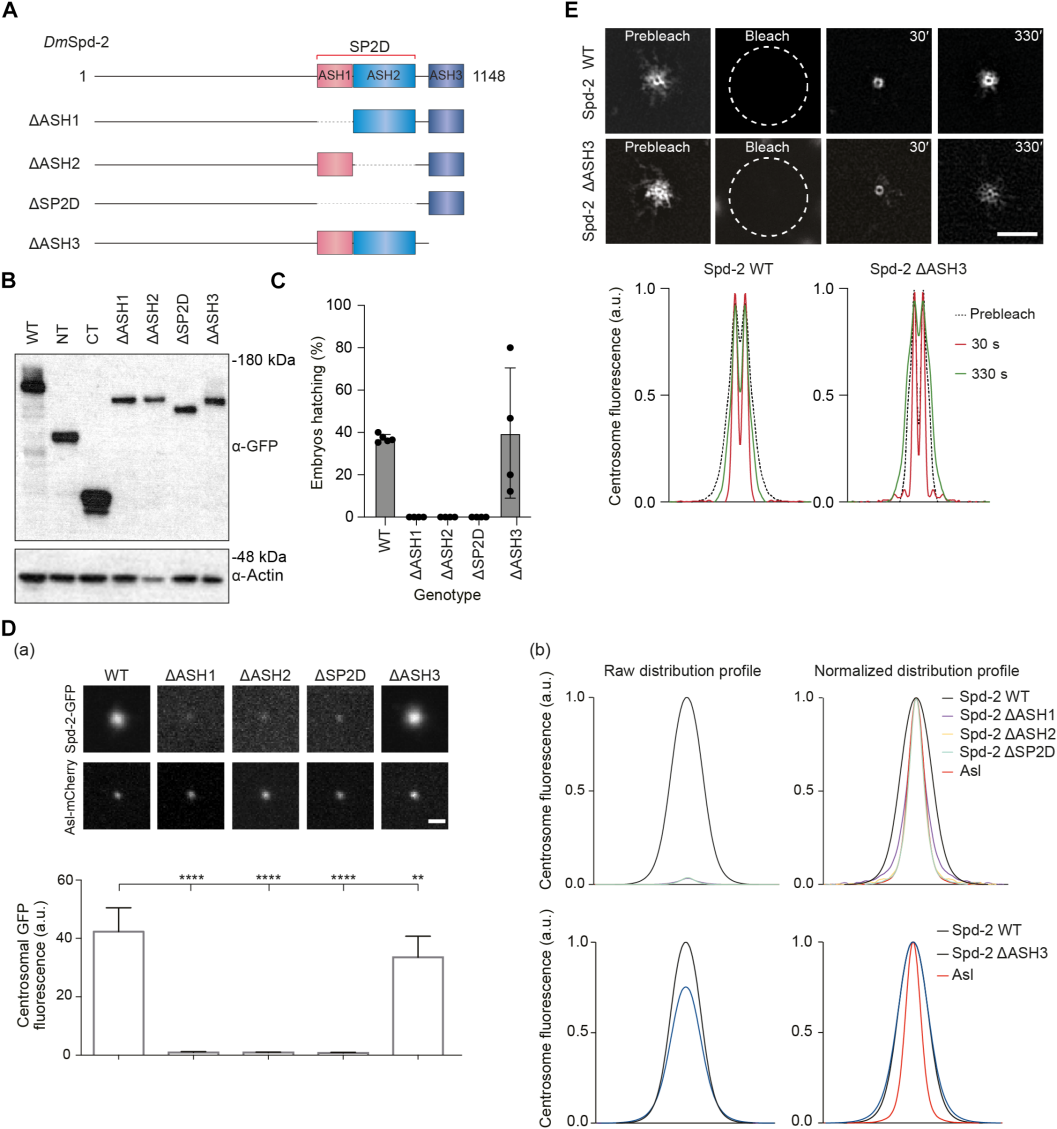
The SP2D, and both its constituent ASH domains, is required for *Dm*Spd-2 scaffold assembly, but ASH3 is not. (**A**) Schematics illustrate the deletion mutants of Spd-2 tested here. (**B**) Western blot shows the expression levels of GFP-fusions to the various deletion mutants shown in (A); actin is shown as a loading control. The expression levels of GFP fusions to the WT and N-terminal and C-terminal truncations (same blot as shown in Fig. 2B) are shown side by side here for ease of comparison. (**C**) Graphs quantify the hatching percentage of embryos laid by *Spd-2^−/−^* females expressing GFP fusions of either WT Spd-2 or the various deletion mutants. *n* = 5 technical repeats; *n* ≥ 100 embryos per technical repeat. (**D**) (a) Confocal images illustrate, and the bar chart below quantifies, the centrosomal fluorescence levels (means ± SD) of the WT and Spd-2 deletion GFP fusions in WT embryos expressing the centriole marker Asl-mCherry injected with mRNA encoding each protein. Statistical significance was assessed using an unpaired *t* test in GraphPad Prism (**P < 0.01 and ****P < 0.0001). (b) Graphs show the raw (left) or normalized (right) centrosomal fluorescence distribution profiles of the WT or Spd-2 deletion GFP fusions. (**E**) 3D-SIM images from a FRAP experiment show the dynamic behavior of WT Spd-2-GFP and Spd-2 ΔASH3-GFP at centrosomes. The time (seconds) after photobleaching is indicated. Graphs show the normalized centrosomal fluorescence distribution profiles of WT Spd-2-GFP and Spd-2 ΔASH3-GFP at successive time points after photobleaching. Scale bars in all images, 2 μm.

To test the role of the individual ASH domains in SPD-2 scaffold assembly, we generated transgenic lines expressing GFP fusions to versions of Spd-2 in which the SP2D (comprising ASH1 + ASH2) or each individual ASH domain was deleted (Fig. 3A). All of these deletion mutants were expressed at similar levels to each other, although at slightly lower levels than WT Spd-2-GFP, Spd-2-NT-GFP, or Spd-2-CT-GFP (Fig. 3B). Fusions in which the SP2D or its constituent individual ASH1 or ASH2 domains were deleted all failed to rescue the sterility of *Spd-2^−/−^* females, but the ASH3 deletion rescued this sterility nearly as well as the WT protein, although more variably (Fig. 3C). The deletion of the SP2D or its constituent individual ASH1 or ASH2 domains markedly reduced the amount of Spd-2 accumulated at centrosomes (Fig. 3D, a) to an even greater extent than the deletion of the entire C-terminal region (Fig. 2). We do not know why this is the case, but this may be at least partially explained by their different expression levels (Fig. 3B).

Unfortunately, the centrosomal levels of these deletion mutants were so low that they could not be analyzed by 3D-SIM, but their normalized distribution profiles suggested that these proteins were still being recruited to centrioles but were not spreading outward to form a scaffold (as their normalized distribution profiles largely overlapped with the Asl-mCherry centriole marker) (Fig. 3D, b). In contrast, Spd-2-∆ASH3-GFP was present at centrosomes at only slightly lower levels than WT Spd-2-GFP, and it appeared to be distributed more evenly between the centrioles and PCM than the WT protein (Fig. 3, D and E). A 3D-SIM FRAP analysis suggested that Spd-2-∆ASH3-GFP was initially recruited to centrioles and then spread outward into the scaffold more quickly than the WT protein (Fig. 3E and movie S2).

Spd-2 plays a major role in mitotic PCM recruitment in flies (*19*, *21*, *39*), so we wanted to test whether the Spd-2 deletion mutants influence the recruitment of the mitotic PCM more generally (and not just the recruitment of Spd-2 itself, as shown above). Unfortunately, females expressing the SP2D, ASH1, or ASH2 deletion mutants in the absence of any endogenous WT Spd-2 lay embryos that fail to develop (Figs. 2C and 3C), so we could only test their effect on PCM assembly by expressing these proteins in embryos that also expressed endogenous WT Spd-2. In this background, the expression of Spd-2-CT, Spd-2-∆ASH1, Spd-2-∆ASH2, or Spd-2-∆SP2D only slightly reduced the centrosomal recruitment of the PCM scaffold protein Cnn, and this was not statistically significant, while the expression of Spd-2-∆ASH3 did not detectably perturb Cnn recruitment (fig. S3). This is not unexpected, as the SP2D, ASH1, and ASH2 deletions are all recruited to centrosomes very poorly (Figs. 2D and 3D), so they presumably cannot effectively displace the endogenous WT Spd-2. The expression of Spd-2-NT, which is still recruited to centrioles but cannot form a scaffold (Fig. 2, D and E) modestly, significantly reduced the centrosomal recruitment of Cnn (fig. S3). This is presumably because Spd-2-NT can better compete with the endogenous WT Spd-2 for recruitment to the centrioles. Collectively, these data indicate that the SP2D and ASH1 and ASH2 are required for Spd-2 and PCM scaffold assembly in vivo, while ASH3 is not.

### Residual Spd-2–dependent scaffold in the absence of Cnn

**Fig. 4.**
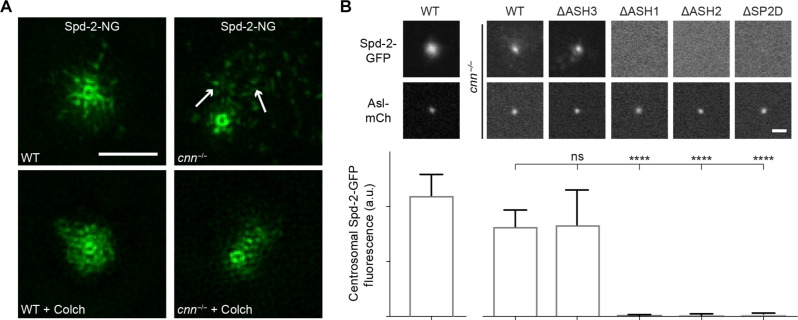
Spd-2 can assemble an SP2D-dependent scaffold structure in the absence of Cnn. (**A**) 3D-SIM images show the behavior of WT Spd-2-NG in *cnn^−/−^* embryos in the presence or absence of colchicine (Colch), which depolymerizes the centrosomal MTs. Arrows highlight small “flares” of Spd-2-NG that rapidly flux away from the centriole on the centrosomal MTs in the *cnn^−/−^* embryos; this outward flux is suppressed if the MTs are depolymerized, and Spd-2-NG forms a scaffold around the centriole in these embryos. (**B**) Confocal images illustrate, and the bar chart below quantifies, the centrosomal fluorescence levels (means ± SD) of WT and Spd-2 deletion GFP fusions in WT embryos (left panels) and *cnn^−/−^* embryos (all remaining panels) expressing the centriole marker Asl-mCherry. Statistical significance was assessed using an unpaired *t* test in GraphPad Prism (****P < 0.0001 and ns, not significant). Scale bars in all images, 2 μm.

How does the SP2D promote PCM scaffold assembly? We reasoned that it might do so in at least two ways, neither of which is mutually exclusive: (i) It might allow Spd-2 to interact with Cnn to promote the assembly of the previously characterized Cnn scaffold (*26*, *39*, *48*). (ii) It might allow Spd-2 to assemble into a scaffold independently of Cnn (either on its own or together with other proteins). To assess these possibilities, we examined whether an Spd-2-mNeonGreen (Spd-2-NG) fusion was capable of forming a scaffold in the absence of Cnn. Cnn does not recruit Spd-2 to centrioles, but it helps maintain Spd-2 at centrosomes (*26*, *39*, *48*). Thus, in embryos laid by *cnn^−/−^* females (hereafter *cnn^−/−^* embryos), the Spd-2-NG scaffold that normally assembles around the centrioles was perturbed, and only small particles of Spd-2-NG could be observed clustered around the centriole (arrows, Fig. 4A, top panels). When the MTs were depolymerized with colchicine, the residual Spd-2-NG scaffold accumulated more prominently around the centriole (Fig. 4A, bottom panels), although, as described previously, the centriole no longer maintained its central position within the PCM—centrioles are well centered in >90% of WT embryos but <10% of *cnn^−/−^* embryos (*49*)—presumably because the PCM scaffold is structurally weakened without Cnn. Thus, Spd-2-NG, either on its own or with other proteins, can form a residual PCM scaffold in the absence of Cnn.

To test whether the SP2D was required for the assembly of this residual, Cnn-independent scaffold, we examined the behavior of the various Spd-2 deletion mutants in *cnn^−/−^* embryos. The deletion of ASH3 did not significantly perturb the assembly of the residual scaffold in *cnn^−/−^* embryos, but the deletion of the SP2D, or of ASH1 or ASH2 individually, appeared to abolish the assembly of this residual scaffold (Fig. 4B). We conclude that the SP2D is required for the assembly of the residual Spd-2 scaffold that can form in the absence of Cnn.

### Structure of the SP2D domain

**Fig. 5.**
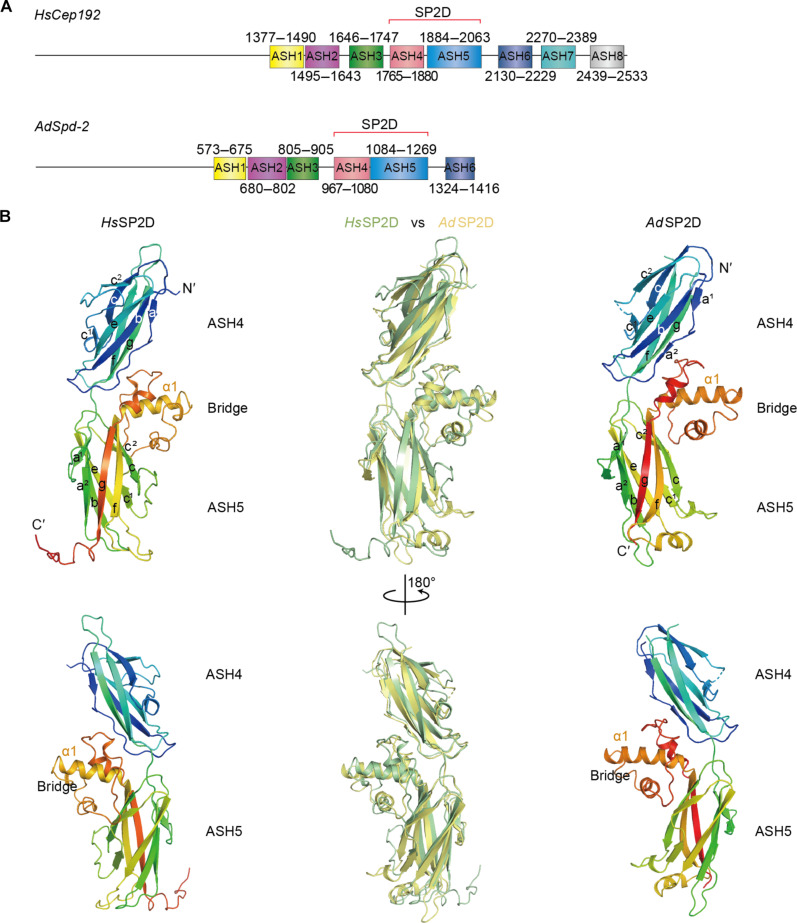
The overall structure of the SP2D is highly conserved. (**A**) Schematics illustrate the domain organization of *H. sapiens* (*Hs*) CEP192 and *A. dorsata* (*Ad*) Spd-2. (**B**) Ribbon representation of two views of the x-ray crystal structures of *Hs*SP2D (containing ASH4 and ASH5) (left panels, green in central overlay panels) and *Ad*SP2D (containing ASH4 and ASH5) (right panels, yellow in central overlay panel). The central α1 helix of the “Bridge” structure—formed by an insertion between strands β-f and β-g in the β-sandwich of the second ASH domain—is highlighted. This structure forms extensive interactions with the first ASH domain that hold the SP2D in its characteristic extended cradle conformation.

We recently reported the x-ray crystal structure of the human SP2D and used this as an example to probe the ability of AF2 to make de novo structural predictions (*50*). The human crystal structure revealed that the two ASH domains of the *Hs*SP2D formed an extended cradle structure and this unique structure was accurately predicted by AF2 (273 Cα pairs with a root mean square deviation (RMSD) of 1.83 Å) (*50*). To experimentally determine whether this structure was conserved in other species, we attempted to solve the structure of the *D. melanogaster* SP2D. Despite extensive efforts, we were unable to crystallize *Dm*SP2D, but we were able to crystallize the SP2D from the honeybee *Apis dorsata* (Fig. 5) and solved its structure to a resolution of 3.5 Å (Table 1). Although side-chain visibility at this resolution is partly limited, the structure allowed its comparison to the human SP2D structure to reveal the underlying conserved interactions (Fig. 5B). Both structures adopt a very similar overall fold (252 Cα pairs with an RMSD of 2.27 Å; Fig. 5B, middle panels). Thus, the extended cradle arrangement of the two ASH domains appears to be a conserved and defining feature of the SP2D.

**Fig. 6.**
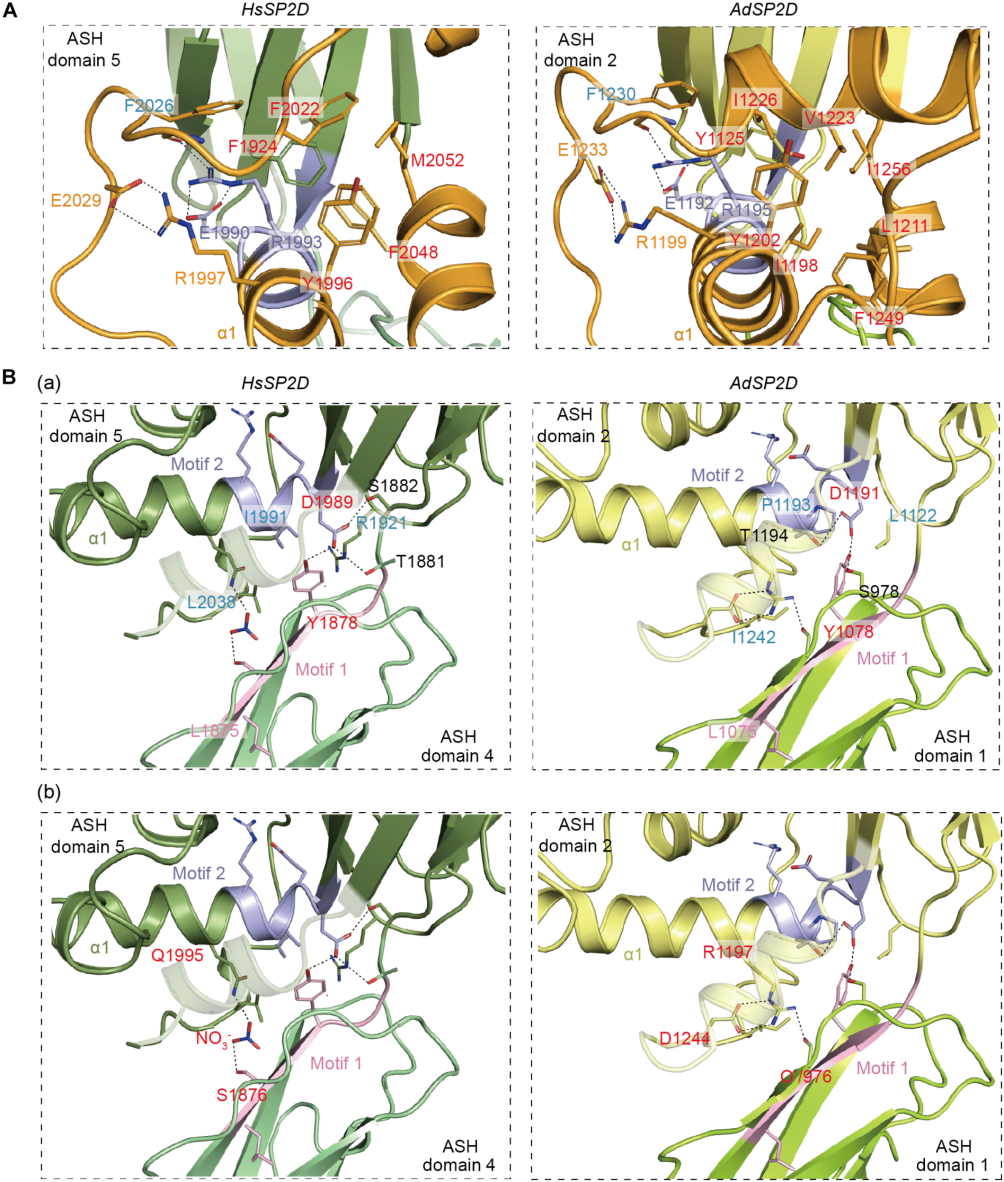
The interactions that maintain the structure of the bridge and the extended cradle of the SP2D are highly conserved. Panels show ribbon and stick representations of the x-ray crystal structures of *Hs* (left panels) and *Ad* (right panels) SP2Ds, highlighting some key interactions that maintain the extended cradle structure. (**A**) View of the interactions within the bridge element that packs between the two ASH domains of SP2D. The bridge is shown in orange, and labeled in red are the residues that form a hydrophobic core around one side of the α1 helix. Further stabilization is provided by a highly conserved network of electrostatic interactions (dotted lines) centered around R1993/R1195 and E1990/E1192 (human/honeybee) in motif 2 (which is highlighted in purple). (**B**) Views on the packing interactions centered around the conserved motifs 1 (pink) and 2 (purple). Interactions discussed in the main text are highlighted in different colors. Note that, although several conserved residues within these motifs contribute to the stability of the structure, the precise interactions they form partly vary between the *Hs*SP2D and *Ad*SP2D structures (see main text for details).

In both humans and honeybees, the extended cradle structure is assembled around a large insertion in the second ASH domain of the SP2D that loops out of the main β-sandwich to form a “bridge” that interacts with the first ASH domain and helps to hold the two ASH domains in their stereotypical conformation (Fig. 5B). This insertion consists of a short helix (α1) that emerges from β-f of the β-sandwich and extends as a less well-structured region that wraps around α1, enclosing a cluster of hydrophobic residues that form a stabilizing core (highlighted in red labels, Fig. 6A). Further stabilization of the bridge structure is provided by a conserved network of electrostatic interactions centered around R1993/R1195 (human/honeybee numbering, used throughout this section) that is located within the evolutionarily conserved motif 2 (GDEXXR; highlighted in purple in Fig. 6A and fig. S4). The side chain of this central Arg is positioned by a salt bridge to the nearby invariant E1990/E1192, also within motif 2, that allows it to form a cation-π interaction with the phenyl ring of F2026/F1230 (light blue label, Fig. 6A), located in the loop region above, and a hydrogen bond to a backbone-carbonyl oxygen in that loop. Another conserved side-chain interaction occurs between R1997/R1199 and E2029/E1233 (orange labels, Fig. 6A). This final pair of contacts assists in positioning the bridge region to stabilize the conformation of the SP2D domain in crystallo.

The stereotypical packing of the two ASH domains of the SP2D is mediated by an interface (~602 Å^2^ for *Hs*SP2D and 736 Å^2^ for *Ad*SP2D) centered around two conserved motifs—motif 1 (LXGYGG) located in β-g of the first ASH domain β-sandwich (pink, Fig. 6B and fig. S4) and motif 2 (GDEXXR) located in the α1 helix of the bridge structure that emerges from the second ASH domain (purple, Fig. 6B and fig. S4). A first set of contacts is based on the conserved Y1878/Y1078 in motif 1 as well as the conserved D1989/D1191 in motif 2 (red labels, Fig. 6B, a), in which the invariant glycines within each motif help to orient, and the invariant leucine within motif 1 that contributes to the hydrophobic core of the first ASH domain. In both structures, the aromatic ring of the Tyr in motif 1 is positioned within a hydrophobic cavity created by side-chain groups in the second ASH domain (L2038, I1991, and R1921 in humans and I1242, P1193, and L1122 in bees) (light blue labels, Fig. 6B, a). In the *Hs*SP2D, the conserved D1989 forms hydrogen bonds with T1881 and S1882 and with the side-chain OH group of the conserved Y1878, whereas in the *Ad*SP2D, the conserved D1191 makes a different set of interactions, forming hydrogen bonds with T1194 within motif 2 and S978 in a loop within the first ASH domain. However, in both structures, these D1989/D1191–based interactions serve the same purpose of orienting the two ASH domains in a similar way.

A second major contact involves electrostatic interactions between the first ASH domain of the SP2D and the α1 helix that extends from the second ASH domain (Fig. 6B, b). As with the first major contact site, the overall architecture of this region is well conserved, but the precise molecular interactions that determine it differ in some details (red labels, Fig. 6B, b). In *Hs*SP2D, Gln^1995^ in the α1 helix forms salt bridge interactions with Ser^1876^ via a nitrate ion trapped in a cavity, whereas in the *Ad*SP2D, Arg^1197^ in the α1 helix forms salt bridge interactions with the side chain of Asp^1244^ and with the backbone-carbonyl oxygen of Trp^976^. Thus, although the stereospecific orientation of the two ASH domains in the SP2D is highly conserved, the internal molecular interactions that ensure their orientation appear to have partly diverged between humans and honeybees.

### In vivo Spd-2 scaffold assembly defects caused by SP2D mutations

**Fig. 7.**
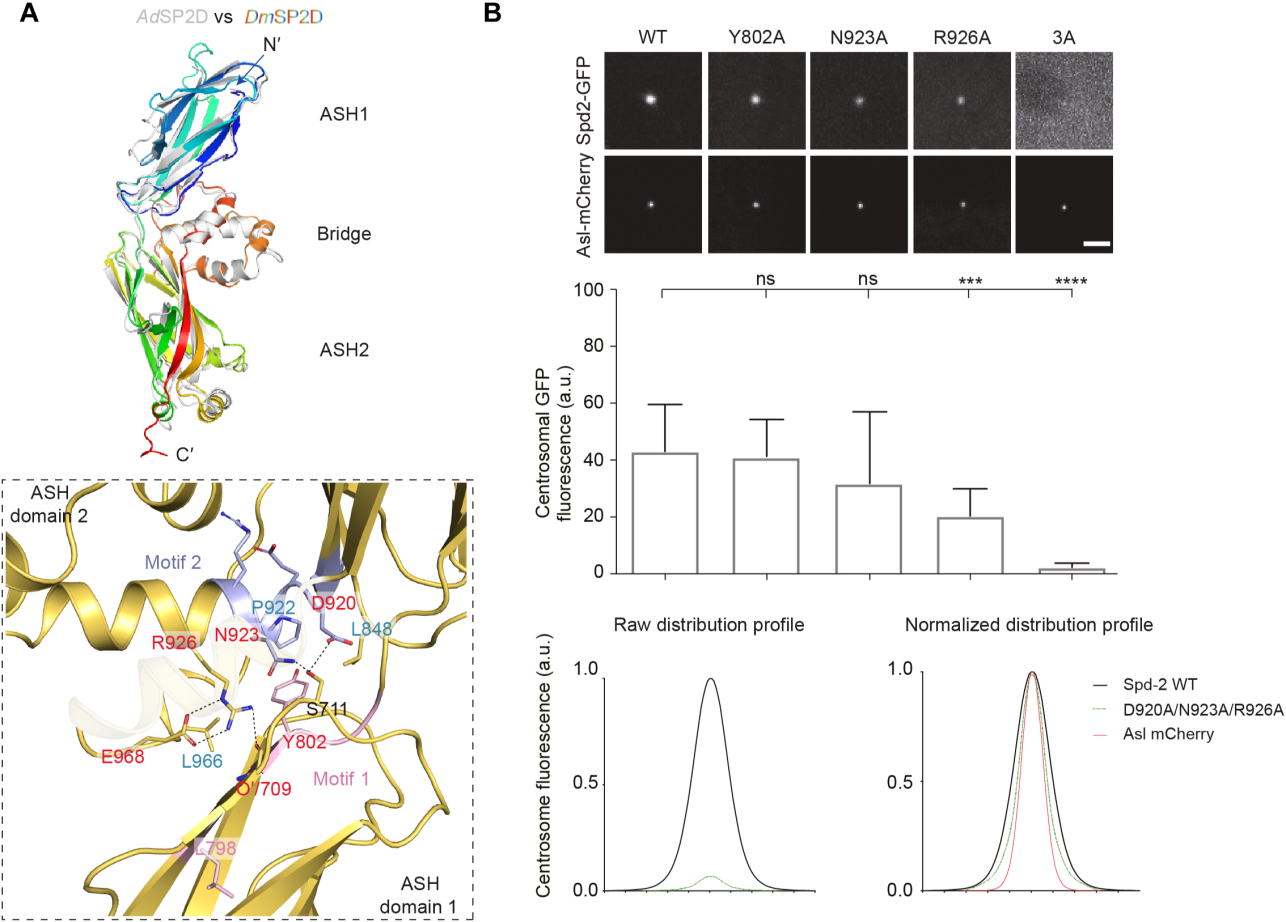
Mutations predicted to disrupt the *Drosophila* SP2D elongated cradle structure perturb Spd-2 scaffold assembly in vivo. (**A**) Ribbon diagram of the AF2-predicted structure of the *D. melanogaster* (Dm) SP2D (rainbow color), overlaid with the *Apis* crystal structure (gray). The inset highlights the core interaction interface that involves hydrophobic (highlighted in blue labels) and electrostatic interactions (dotted lines) from motifs 1 and 2, which are similar to the stabilizing interactions observed in the *Apis* structure (Fig. 6B). (**B**) (a) Confocal images illustrate, and the bar chart below quantifies, the centrosomal fluorescence levels (means ± SD) of the WT and various Spd-2 mutant GFP fusions in WT embryos expressing the centriole marker Asl-mCherry and injected with mRNA encoding each protein. Statistical significance was assessed using an unpaired *t* test in GraphPad Prism (***P < 0.001 and ****P < 0.0001; ns, not significant). (b) Graphs show the raw (left) or normalized (right) centrosomal fluorescence distribution profiles of the WT or Spd-2-3A mutant (D920A/N923A/R926A) GFP fusions. Scale bar, 2 μm.

To test the significance of the stereospecific orientation of the SP2D, we used AF2 to predict the structure of the *Dm*SP2D. The overall fold of the predicted structure was similar to the *Hs*SP2D and *Ad*SP2D, and the key molecular interactions maintaining the orientation of the predicted *Dm*SP2D were most similar to those seen in the *Ad*SP2D crystal structure (Fig. 7A and fig. S5).

We then generated individual alanine substitutions of several of the amino acids predicted to be in the major contact interface (inset, Fig. 7A): Y802, which is buried in the interface but does not form specific electrostatic interactions within it; N923, which is predicted to form a single electrostatic bond with S711; and R926, which appears to be central to the interface, as it forms three electrostatic bonds: two with E968 and one with a backbone-carbonyl oxygen. These alanine substitutions affected full-length Spd-2-GFP scaffold assembly in vivo in a graded manner that correlated with the number of electrostatic bonds those residues formed in the predicted interaction interface: Y802A had no effect, and N923A had a mild but statistically insignificant effect, while the R926A mutation significantly perturbed Spd-2 scaffold assembly (Fig. 7B). Circular dichroism (CD) analysis of purified *Dm* SP2D-R926A showed it to be largely folded as a β sheet, indicating that the mutation did not strongly perturb the overall fold of the SP2D (fig. S6). We also generated a triple alanine substitution (3A) of three amino acids predicted to make key hydrogen bonding or electrostatic interactions in the SP2D domain packing interface (D920A, N923A, and R926A). These mutations essentially abolished the ability of full-length Spd-2-GFP-3A to form a scaffold in vivo (Fig. 7B), but we note that purified SP2D-3A was largely insoluble in vitro, suggesting that this combination of mutations probably perturbs the overall fold of the SP2D. Nevertheless, collectively, these results suggest that the conserved stereotypical arrangement of the SP2D is important for Spd-2’s function in promoting PCM scaffold assembly.

**Fig. 8.**
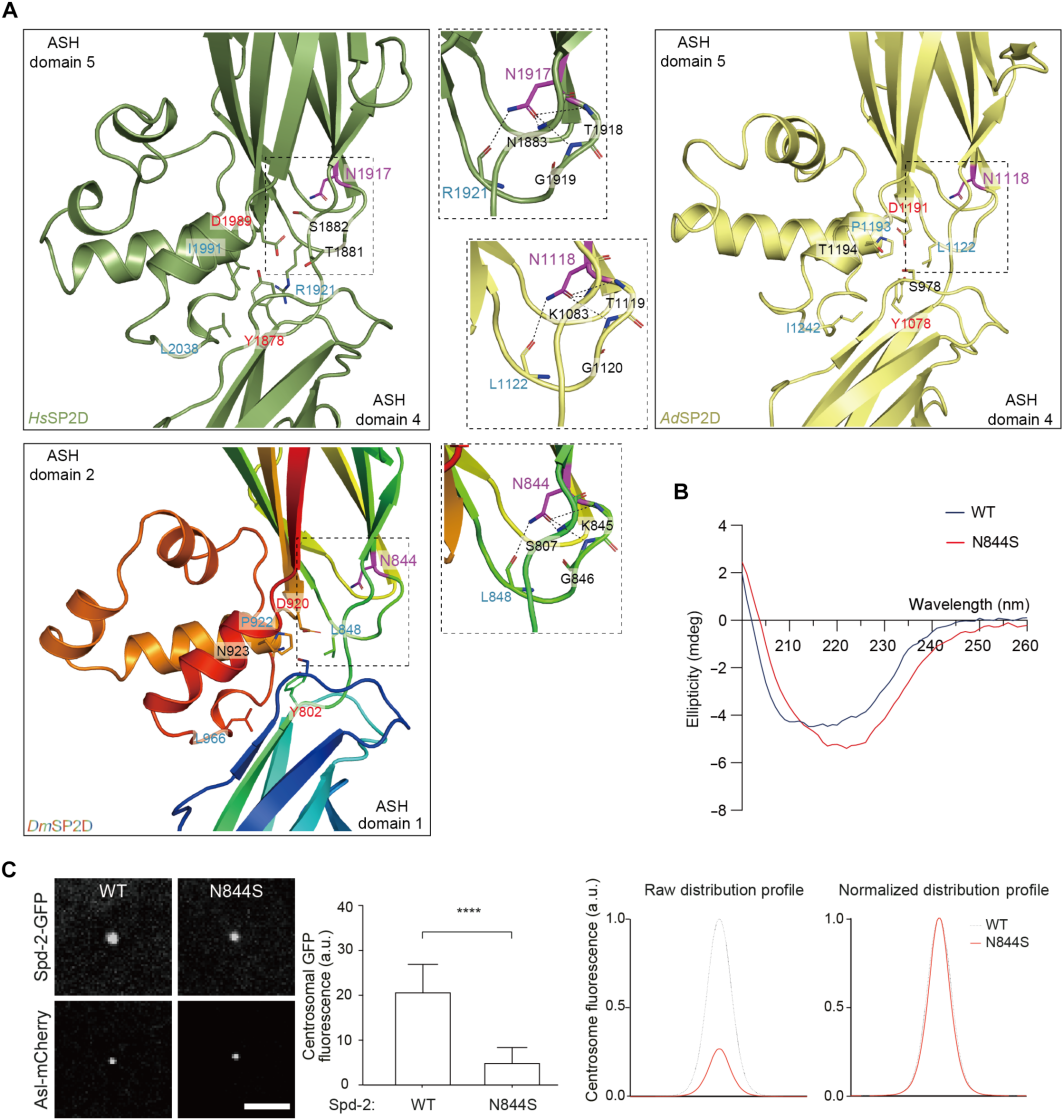
A human disease substitution (N1917S) located near the interaction interface between the two ASH domains of SP2D mildly perturbs *Drosophila* Spd-2 scaffold assembly in vivo. (**A**) Views of the interaction network (dotted lines) made by the side chain of the conserved Asn1917/Asn1118/Asn844 (labeled in magenta) in humans, *Apis*, and *Drosophila*, respectively, that is substituted for a Ser in human patients. (**B**) CD analysis of *Dm*SP2D WT and N844S shows that this substitution appears to slightly alter the protein fold. (**C**) (a) Confocal images illustrate, and the bar chart quantifies, the centrosomal fluorescence levels (means ± SD) of WT and Spd-2-N844S mutant GFP fusions in WT embryos expressing the centriole marker Asl-mCherry and injected with mRNA encoding each protein. Statistical significance was assessed using an unpaired *t* test in GraphPad Prism (****P < 0.0001). (b) Graphs show the raw (left) or normalized (right) centrosomal fluorescence distribution profiles of the WT and Spd-2-N844S GFP fusions. Scale bar, 2 μm.

It has recently been found that mutations in human CEP192 can lead to MVA syndrome with tetraploidy and also a predisposition to male infertility (*41*). Male infertility was associated with a monoallelic Asn1917Ser substitution, while MVA and tetraploidy were associated with a biallelic variant containing the Asn1917Ser variant together with an additional His638Try variant. N1917—and its equivalents in *Apis* (N1118) and *Drosophila* (N844)—forms part of the interaction interface between the two ASH domains of the SP2D (magenta labels, Fig. 8A) and makes interactions that help stabilize it (inset, Fig. 8A). To test the potential significance of this mutation in vivo, we made the equivalent substitution in *Dm*SP2D (N844S). This mutant protein exhibited reduced solubility compared to the WT protein in vitro, suggesting that the protein fold was, at least partly, disrupted, an observation confirmed by CD spectroscopy (Fig. 8B). A full-length Spd-2-N844S-GFP mutant exhibited significantly reduced, but not abolished, centrosomal localization in vivo (Fig. 8C)—potentially explaining why this substitution is pathological in humans.

### Potential Spd-2 self-interaction interface

**Fig. 9.**
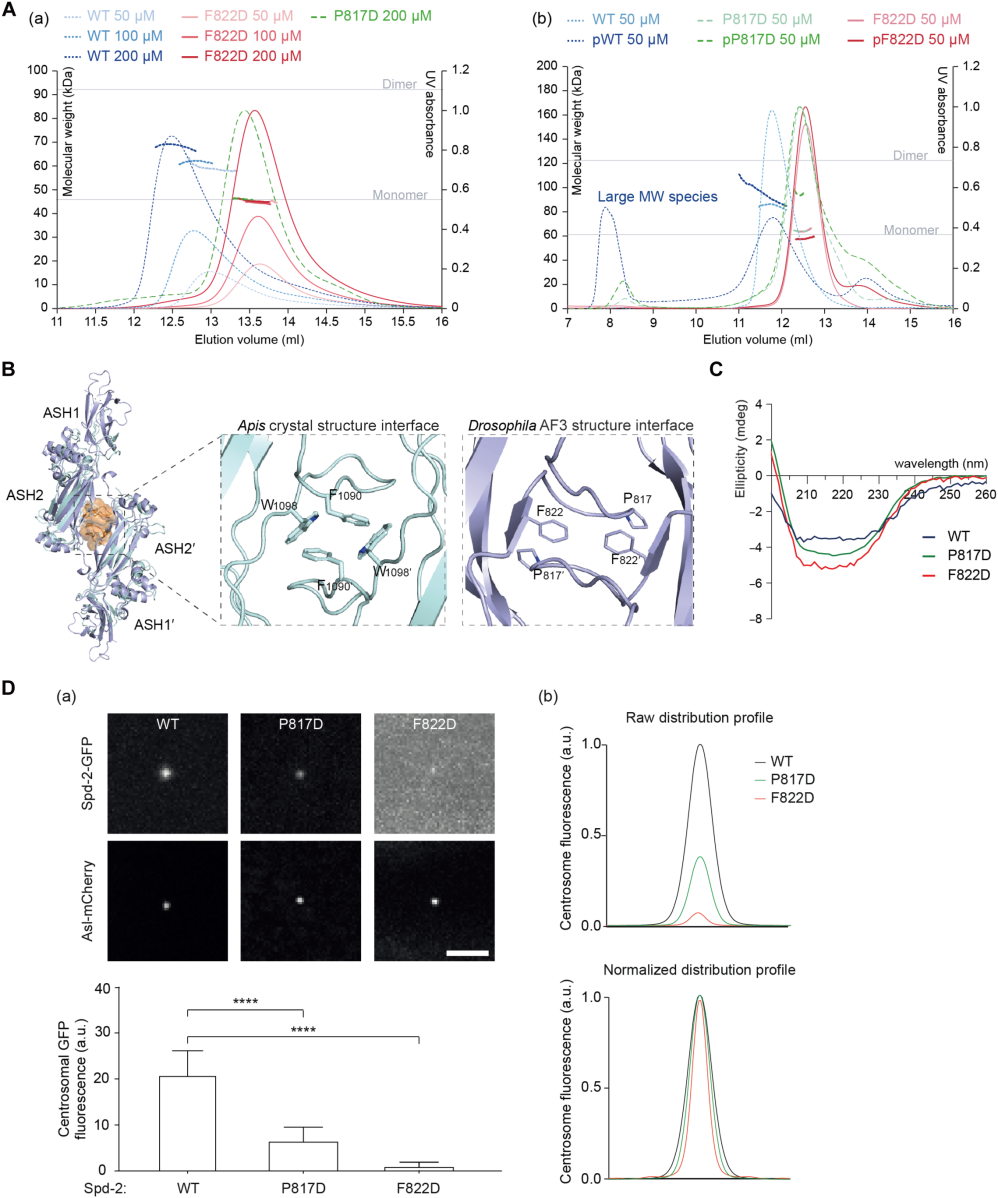
A hydrophobic interaction interface may allow insect SP2Ds to form dimers. (**A**) (a) SEC-MALS analysis of Sumo-tagged SP2D WT, P817D, or F822D performed at a range of protein concentrations (as indicated). Note how the WT, but not mutant, protein(s) has/have a tendency to form higher MW species at higher concentrations. (b) SEC-MALS analysis of Sumo-tagged Spd-2-CT (697 to 1146 amino acids) WT, P817D, or F822D that has been phosphorylated in vitro with recombinant human PLK1 kinase. The orange horizontal dotted lines indicate the theoretical mass of a monomer or dimer. Note how the WT, but not mutant, protein(s) can form large species when phosphorylated by PLK1. (**B**) Ribbon diagram showing the packing interactions in the top-ranked AF3 prediction of a *Dm*SP2D dimer (purple) overlaid with the *Ad*SP2D dimer (cyan) observed in crystallo; a similar hydrophobic interface observed in both structures is highlighted in orange and is shown in detail in the inset. (**C**) CD analysis of SP2D WT, P817D, or F822D shows that each single-point mutation does not strongly affect the protein fold. (**D**) (a) Confocal images illustrate, and the bar chart below quantifies, the centrosomal fluorescence levels (means ± SD) of WT and Spd-2-P817D or F822D mutant GFP fusions in WT embryos expressing the centriole marker Asl-mCherry and injected with mRNA encoding each protein. Statistical significance was assessed using an unpaired *t* test in GraphPad Prism (****P < 0.0001). (b) Graphs show the raw (top) or normalized (bottom) centrosomal fluorescence distribution profiles of the WT and Spd-2-P817D or F822D GFP fusions. Scale bar, 2 μm.

The *Dm*SP2D protein exhibited a slight tendency to form dimers in solution at high concentrations (blue curves, Fig. 9A, a), and we noticed that the entire C-terminal half of *Dm*Spd-2 (Spd-2^697–1146^, containing all three ASH domains) could assemble into large-molecular-weight species when it was phosphorylated by recombinant human PLK1 in vitro (dark blue curve versus light blue curve, Fig. 9A, b). This raised the intriguing possibility that phosphorylation by Polo/PLK1 might allow Spd-2 molecules to assemble into higher-order scaffolds in vivo. We used AF3 to screen for potential dimer interactions between the C-terminal half of Spd-2, and the top five solutions all predicted a highly similar dimer configuration {light blue, Fig. 9B [the predicted template modeling (pTM) score, 0.72; the interface predicted template modeling (ipTM) score, 0.61] and fig. S7} that was centered around packing interactions between P817 and F822. Intriguingly, a similar crystal-packing interaction interface involving F1090 and W1098 was observed in the *Apis* Spd-2 SP2D crystal structure (cyan, Fig. 9B).

To examine the potential relevance of these crystal/predicted contacts, we substituted charged amino acids in place of the hydrophobic contact amino acids in the *Drosophila* protein (P817D and F822D). Neither substitution appeared to markedly perturb the folding of the SP2D domain in vitro (Fig. 9C), but both substitutions strongly perturbed the ability of the C-terminal region of Spd-2 to form high-molecular-weight complexes when phosphorylated by PLK1 in vitro (red and green curves, Fig. 9A, b). Perhaps unexpectedly, both point mutations also strongly perturbed the ability of full-length Spd-2-GFP to form a centrosome scaffold in vivo—with the F822D substitution perturbing scaffold assembly to a similar extent as deleting the entire SP2D (Fig. 9D). These results suggest that this putative Spd-2 self-interaction interface might be important for Spd-2 scaffold assembly in vivo.

## DISCUSSION

Spd-2/CEP192 proteins play a central part in mitotic centrosome assembly, and the SP2D is the defining feature of this family of proteins. Our crystallography studies show that, in humans and honeybees, the SP2D adopts an extended cradle conformation, with two ASH domains held together by an extensive interaction interface centered around two of the most highly conserved motifs—motif 1 (PLXGYGG) and motif 2 (GDEXXR). These crystal structures were not present in the Protein Data Bank (PDB) at the time AF2 was released, yet AF2 accurately predicted this conserved stereotypical arrangement in human CEP192 (*50*), and it predicts a similar arrangement for the SP2D in Spd-2/CEP192 proteins from several other species, including *Drosophila* and *Caenorhabditis elegans* (*43*, *51*). Although several centrosome, cilia, flagella, and Golgi complex proteins contain ASH domains (*40*), to our knowledge, the extended cradle structure of the SP2D has not been observed in any other ASH-domain proteins. Our studies in *Drosophila* reveal that mutations predicted to perturb the interface between the ASH domains can suppress Spd-2’s ability to promote PCM scaffold assembly.

These findings strongly suggest that the stereotypical extended cradle structure of the SP2D is central to Spd-2/CEP192’s scaffolding function. A priori, it would seem likely that the primary function of the SP2D is to specifically interact with other proteins to initiate mitotic PCM scaffold assembly. If so, one might expect there to be several conserved regions on the external surface of the SP2D, yet unexpectedly, the most conserved regions of the SP2D (motifs 1 and 2, fig. S4) seem to be involved in the internal interactions that maintain the specific orientation of the two ASH domains. Outside of these regions, there is very little conservation between, for example, insects and vertebrates (fig. S4). Thus, it remains unclear why the SP2D fold is so conserved and how this stereospecific structure contributes to mitotic PCM assembly.

Our previous study in fly embryos indicated that *Drosophila* Spd-2 can form a residual PCM scaffold that can recruit small amounts of mitotic PCM even in the absence of Cnn (*39*). Here, we show that the assembly of this residual PCM scaffold is dependent on the SP2D domain. The fly SP2D has a weak tendency to dimerize at higher concentrations, and the C-terminal half of *Dm*Spd-2 can form much larger species in vitro if it is phosphorylated by recombinant PLK1. This suggests that phosphorylated Spd-2 may be able to homo-oligomerize to form higher-order assemblies, perhaps explaining how it can form a residual scaffold in the absence of Cnn. Although caution should be taken when extrapolating in vivo protein behavior from the behavior of isolated proteins in vitro, it is intriguing that this in vitro behavior mimics well the situation in vivo, where Spd-2’s ability to promote PCM scaffold assembly depends on its phosphorylation (*21*, *26*). On the other hand, we have recently shown that *Drosophila* Spd-2 can stimulate the assembly of a second, Cnn-independent, PCM scaffold by recruiting Aurora A, which phosphorylates transforming acidic coiled-coil-containing protein (TACC) to form a scaffold that can recruit many other PCM proteins (*52*). This mechanism would also allow Spd-2 to form a residual PCM scaffold in the absence of Cnn.

In relation to this point, it is intriguing that single substitutions in two amino acids (P817D and F822D) that are predicted to be involved in a potential *Dm*Spd-2 dimerization interface (that is similar to a crystal-packing interface present in the *Ad*SP2D crystal structure) strongly suppress the assembly of PLK1-dependent SP2D higher-order assemblies in vitro and also Spd-2 scaffold assembly in vivo. This is consistent with the possibility that phosphorylated Spd-2 may itself be able to form higher-order assemblies that are relevant for PCM scaffold assembly. Alternatively, perhaps the P817D and F822D mutations perturb interactions with other proteins required for mitotic PCM scaffold assembly, such as Cnn or TACC (*52*). It is also worth noting that the precise function of Spd-2 in mitotic PCM assembly may vary between species and perhaps even between cell types. For example, while Cnn is clearly not required to recruit Spd-2 to centrosomes in fly embryos (*39*), it has been reported that Cnn can recruit Spd-2 to centrosomes in fly cultured cells (*12*).

Spd-2/CEP192 molecules in some species contain only two ASH domains that form the SP2D, but in many species, these molecules contain additional ASH domains (e.g., bees have four, humans have six, and flies have one ASH domain in addition to the SP2D domain). These additional ASH domains presumably provide protein-interaction modules, but their functions are unknown. In flies, the single additional ASH domain is not essential for the Spd-2 function (as Spd-2-∆ASH3-GFP can rescue the female sterility of *Spd-2* mutants) nor for Spd-2 scaffold assembly or for targeting Spd-2 to the centriole/centrosome. Instead, deletion of ASH3 subtly changes the centrosomal distribution of Spd-2, with Spd-2-∆ASH3-GFP being recruited to lower levels than WT but distributed more evenly between the mother centriole and the mitotic PCM. Clearly, more experiments are required to understand the basis of this phenotype.

Last, given that Spd-2/CEP192 molecules play such a central part in centrosome biology, it is perhaps unexpected that the most conserved part of these molecules, the SP2D, is not required to target these molecules to centrosomes. Instead, our studies indicate that the N-terminal half of Spd-2 is sufficient for targeting to the centriole. Once targeted to the centriole, however, Spd-2 molecules lacking the SP2D cannot incorporate into the PCM. The N-terminal regions of Spd-2/SPD-2/Cep192 in flies/worms/humans appear to be largely unstructured, with AlphaFold predicting only a small number of low-confidence short helical regions. In flies, the centriole protein Ana1 (*53*)—human CEP295 (*54*–*56*)—appears to play an important part in recruiting Spd-2 to centrioles (*26*), so it will be important to determine whether, and how, Ana1 interacts with the N-terminal region of Spd-2 and whether these interactions are conserved in humans.

## MATERIALS AND METHODS

### Fly husbandry, stocks, and handling

Flies were kept at 25° or 18°C on *Drosophila* culture medium (0.77% agar, 6.9% maize, 0.8% soya, 1.4% yeast, 6.9% malt, 1.9% molasses, 0.5% propionic acid, 0.03% *o*-phosphoric acid, and 0.3% nipagin). The following fly lines have been previously described: Ubq-Spd-2-GFP (*19*), Ubq-Spd-NG (*52*), and Asl-mCherry (*57*). The GFP-Ubq-Spd-2 mutant transgenic lines were generated by the Fly Facility in the Department of Genetics, Cambridge (UK), via random P-element insertion into a *w^1118^* background; *w^1118^* flies were used as WT controls. Embryos were collected on cranberry-raspberry juice plates (25% cranberry-raspberry juice, 2% sucrose, and 1.8% agar) supplemented with fresh yeast. Standard fly handling techniques were used (*58*).

In vivo studies were performed using 1.5- to 2-hour-old syncytial blastoderm stage embryos. After 0- to 1-hour collections at 25°C, embryos were aged at 25°C for 30 to 60 min. When injecting mRNA, embryos were collected for 30 min, injected, and imaged after 90 min at 21°C (always at the syncytial blastoderm stage). Before injection or imaging, embryos were dechorionated on double-sided tape and mounted on a strip of glue onto a 35-mm glass-bottom petri dish with a 14-mm microwell (MatTek). After desiccation for 1 min (noninjection experiments) or 6 min (pre-mRNA injection) at 25°C, embryos were covered in Voltalef oil (ARKEMA). When analyzing the effect of MT depolymerization, embryos were first injected with a 1 mM colchicine solution and imaged 20 to 60 min later.

### Hatching rate analysis

Embryos were collected for 1 to 5 hours and then aged for 24 hours. The percentage of embryos that had hatched out of their chorion was calculated.

### In vitro mRNA production and microinjection

The mRNA injection assay has been described previously (*59*). All Spd-2 mutations were generated on a plasmid containing Spd-2 cDNA fused to GFP (pRNA-Spd-2-GFP). Point mutations were generated by site-directed mutagenesis using the Q5 Site-Directed Mutagenesis Kit (NEB) on the WT cDNA.

mRNA was synthesized in vitro using an mMESSAGE mMACHINE T3 Transcription kit (Thermo Fisher Scientific; AM1348), and RNA was purified using an RNeasy MinElute kit (Qiagen; 74106) according to the manufacturer’s instructions. All RNA constructs were stored at −80°C and injected at a concentration of 2 mg/ml.

### Spinning disk confocal microscopy

Embryos were imaged at 21°C essentially as described previously (*60*) on a Perkin Elmer ERS spinning disk (Volocity software version 6.3) mounted on a Zeiss Axiovert 200M microscope using a 63×/1.4–numerical aperture (NA) oil immersion objective and an Orca ER CCD camera (Hamamatsu Photonics) (Figs. 2D, 3B, and 4B) or on an Andor Revolution system equipped with an EM-CCD Andor iXon+ camera on a Nikon Eclipse TE200-E microscope using a Plan-Apochromat 60×/1.42-NA oil differential interference contrast lens, controlled with Andor Fusion software (Figs. 7B, 8C, and 9D). Confocal sections of 11 slices (23 slices for Fig. 8C) with 0.5-μm-thick intervals were collected with 488- and 568-nm lasers used to excite GFP and mCherry respectively.

ImageJ was used to calculate the centrosomal fluorescence intensity profile of the different Spd-2-GFP proteins, as described previously. The center of mass of the centrosome was calculated by thresholding the image and running the “analyse particles” (center of mass) macro on the most central *z* plane of the centrosome. Centered concentric rings (spaced at 0.028 μm and spanning across 3.02 μm) were created around this center, and the “raw” average fluorescence intensity around each ring was measured (using the radial-profiling function). After subtracting the average cytosolic signal (background), we normalized the data so that the peak intensity of the prebleached image was equal to 1. All intensity profiles were “mirrored” so that they show a full symmetric profile centered around the center of the centrosome. Five centrosomes from at least seven embryos were used to calculate the average distribution for each protein type.

### 3D-SIM

3D-SIM microscopy was performed essentially as described previously (*39*) on an OMX V3 Blaze microscope (GE Healthcare, Micron Oxford) with a 60×/1.42-NA oil UPlanSApo objective (Olympus), 488- and 593-nm diode lasers, and Edge 5.5 scientific complementary metal-oxide semiconductor cameras (PCO). Raw acquisition was reconstructed using softWoRx 6.1 (GE Healthcare) with a Wiener filter setting of 0.006 and channel-specific optical transfer functions. Living embryos were imaged at 21°C, acquiring stacks of six *z*-slices (0.125-μm intervals). Spherical aberration was minimized by matching the refractive indices (1.514) of the immersion oil to the sample. The images shown are maximum intensity projections. The images from the different color channels were registered with alignment parameters obtained from calibration measurements using 1- to 0.2-μm TetraSpeck Microspheres (Thermo Fisher Scientific) using Chromagnon alignment software (*61*). The SIMcheck plug-in in ImageJ (NIH, Bethesda, MD) was used to assess the quality of all SIM reconstructions (*62*). To perform 3D-SIM FRAP, we used the software development kit from GE Healthcare, as described previously (*57*).

The centrosomal profiles were calculated in a similar way to that described above, except that the concentric rings for Asl-GFP and Spd-2-GFP were spaced at 0.0055, 0.011, and 0.0109 μm and spanned across 1.86, 3.28, and 3.28 μm, respectively. For generating the average 3D-SIM profiles for Asl-GFP and Spd-2-GFP, we averaged profiles from 11 and 15 centrosomes, respectively.

### Western blot analysis

Western blotting was performed as described previously (*60*). The following primary antibodies were used at 1/500 dilution: mouse-anti-GFP (Roche; RRID: AB_390913) mouse anti-Actin (Sigma-Aldrich; RRID: AB_476730). For visualization, we used the SuperSignal West Femto kit (Thermo Fisher Scientific; 34095) and appropriate horseradish peroxidase–conjugated secondary antibodies at 1/3000 dilution (GE Healthcare, NA931V).

### Recombinant protein expression and purification

#### Drosophila *ASH1*

The cDNA sequence encoding *Drosophila* Spd-2^697–805^ (ASH1 domain) was subcloned into a pETM14 (EMBL) vector encoding an N-terminal His_6_ tag. Protein was expressed in *Escherichia coli* (*E. coli*) B834 (DE3) strains in LB broth at 21°C and purified using Ni-NTA chromatography followed by size exclusion chromatography [50 mM tris-HCl (pH 7.5), 150 mM NaCl, and 5 mM β-mercaptoethanol]. ^15^N-^13^C double-labeled ASH1 protein was obtained by growing bacteria in M9 minimal medium using ^13^C glucose and ^15^N ammonium chloride (Sigma-Aldrich) as the only carbon and nitrogen sources, respectively. N-terminal His_6_ tag was cleaved off using GST-3C protease and dialyzed into 50 mM tris-HCl (pH 7.5), 150 mM NaCl, and 5 mM β-mercaptoethanol at 4°C overnight. The untagged protein was further purified via size exclusion chromatography [50 mM NaH_2_PO_4_/Na_2_HPO_4_ (pH 7.0), 150 mM NaCl, and 2 mM dithiothreitol (DTT)].

#### Drosophila *ASH3*

The cDNA sequence encoding *Drosophila* Spd-2^1047–1146^ (ASH3 domain) was subcloned into a pETM14 (EMBL) vector encoding an N-terminal His_6_ tag. Protein was expressed in *E. coli* B834 (DE3) strains in LB broth at 21°C and purified using Ni-NTA chromatography followed by size exclusion chromatography [50 mM tris-HCl (pH 7.5), 150 mM NaCl, and 5 mM β-mercaptoethanol]. For size exclusion chromatography-multiangle light scattering (SEC-MALS) analysis and crystallization trials, the N-terminal His tag was cleaved off using GST-3C protease and dialyzed into 50 mM tris-HCl (pH 7.5), 150 mM NaCl, and 5 mM β-mercaptoethanol at 4°C overnight. The untagged protein was further purified via size exclusion chromatography [50 mM tris-HCl (pH 7.5), 150 mM NaCl, and 1 mM tris(2-carboxyethyl)phosphine hydrochloride (TCEP)].

#### Human ASH7

DNA encoding human CEP192^2256–2402^ (numbering based on UniProt Q8TEP8) was cloned into a pET28-derived vector to create an open reading frame with an N-terminal His_6_ tag that can be removed by cleavage with PreScission protease. The construct was expressed in *E. coli* Rosetta (DE3) cells at 18°C in supplemented M9 medium as described (*63*) to produce recombinant SeMet CEP192^2256–2402^. Subsequently, the protein was purified from cell lysates by Ni-NTA (Qiagen) chromatography. The Ni-NTA eluate was dialyzed in 10 mM tris-Cl (pH 8.0), 50 mM NaCl, and 1 mM DTT, and the tag was cut off with PreScission protease. Subsequently, the protein was further purified by size exclusion chromatography in 10 mM tris-Cl (pH 8.0), 50 mM NaCl, and 1 mM DTT followed by ion-exchange chromatography (HiTrap Q FF, GE Healthcare) and eluted by a linear salt gradient from 10 mM tris-Cl (pH 8.0), 2 mM DTT (buffer A) to buffer A supplemented with 1 M NaCl. The eluted protein was concentrated, snap frozen in liquid nitrogen, and stored at −80°C.

#### Drosophila *SP2D*

The cDNA sequence encoding *Drosophila* Spd-2^697–998^ (the SP2D domain) WT or P817D or F822D mutants was subcloned into a pET28a (Novagen) vector encoding an N-terminal His_6_-Sumo tag. Proteins were expressed in *E. coli* BL21 (DE3) strains in LB broth at 16°C and purified using Ni-NTA chromatography followed by size exclusion chromatography [50 mM tris-HCl (pH 8.0), 300 mM NaCl, 10% glycerol, and 1 mM DTT]. The N-terminal Sumo tag was cleaved off using Ulp1 protease at 4°C overnight. The untagged protein was further purified via reverse Ni-NTA chromatography and size exclusion chromatography [50 mM NaH_2_PO_4_/Na_2_HPO_4_l (pH 7.0), 300 mM NaCl, 10% glycerol, and 1 mM DTT].

To purify the entire C-terminal half of Spd-2 protein (Spd-2^697–1146^), we used sortase A–mediated protein ligation method to fuse Spd-2^697–1045^–LPETGG with Spd-2^1046–1146^. The two protein fragments that are to be ligated were incubated at 1:4 molar ratios at room temperature for 4 hours in the presence of 100 mM CaCl_2_. To terminate the ligation reaction and to purify the ligated protein product, the reaction was subjected to further purification via size exclusion chromatography [50 mM tris-HCl (pH 8.0), 300 mM NaCl, 10% glycerol, and 1 mM DTT].

#### Honeybee SP2D

DNA encoding *A. dorsata* CEP192^946–1284^ (numbering based on *A. dorsata* protein LOC102681909 isoform X1; accession number: XP_006619497.1) was cloned into vector pACE Bac1 and was N-terminally tagged with the His_6_-lipoyl domain from *Bacillus stearothermophilus* dihydrolipoamide acetyltransferase (*64*). The tag can be removed by cleavage with PreScission protease, leaving the amino acid residues GP on the N terminus. Baculoviruses were obtained from this construct using standard procedures and used to infect *Sf*9 insect cell suspension cultures in ESF921Δ, methionine-deficient medium (Oxford Expression Technologies) at a cell density of ~1 × 10^6^ cells/ml at 27°C. Ten, 23, 38.5, 48, and 63 hours postinfection, l-selenomethionine was added to the culture to a concentration of 0.05 mg/ml and the cells were harvested 72 hours postinfection. Proteins were purified from cell lysates by Ni-NTA (Qiagen) chromatography. Purified GST-PreScission protease was added, and the eluates were dialyzed against 50 mM tris-Cl (pH 8.0), 500 mM NaCl, 5 mM imidazole (pH 7.6), and 10 mM β-mercaptoethanol. The cleaved eluate was incubated with Ni-NTA (Qiagen) resin, and the flowthrough was purified further by gel filtration on a Sephacryl S-300 column (GE Healthcare) run in 10 mM tris-Cl (pH 8.0), 500 mM NaCl, and 4 mM DTT. Peak fractions were concentrated, snap frozen in small aliquots, and stored at −80°C.

### Protein crystallization and structure determination

#### Drosophila *ASH3*

Crystals of *Drosophila* Spd2^1047–1146^ protein were obtained by mixing the freshly purified protein at 52 mg/ml with equal volumes of reservoir solution containing 100 mM Hepes (pH 7.5), 2 M ammonium sulfate, and 5% (w/v) polyethylene glycol, molecular weight 400 (PEG-400) using the sitting-drop vapor diffusion method at 20°C. Before diffraction experiments, 20% (v/v) ethylene glycol was added as the cryoprotectant. A 1.93-Å-resolution dataset was collected at the I04-1 beamline at Diamond Light Source at a wavelength of 0.92 Å. Data were processed using Xia2 pipeline (*65*) in the 3daii mode (using XDS) (*66*) and AIMLESS (*67*) and were indexed to the space group *I*4_1_22. The phase problem was solved by molecular replacement using a polyalanine model derived from the structure of the human VAPB MSP domain (PDB code: 3IKK) with MOLREP in CCP4 (*68*). Manual model building and refinement were performed iteratively using Coot (*69*) and Refmac5 (*70*). The final model was validated by MolProbity (*71*), and statistics are summarized in Table 1.

#### Honeybee SP2D and human ASH7

SeMet human CEP192^2256–2402^ crystals were obtained from a hit in the MemSys screen [Molecular Dimensions (MD1-25); reservoir solution: 100 mM Na-citrate (pH 5.5), 30% PEG-400, 100 mM NaCl, and 100 mM MgCl_2_] by the vapor diffusion method at 19°C using a 100-nl protein solution and a 100-nl reservoir solution. Crystals were mounted in the mother liquor after 1 day and frozen in liquid nitrogen.

SeMet *A. dorsata* CEP192^946–1284^ crystals were obtained by the vapor diffusion method at 19°C using a 100-nl protein solution and a 100-nl reservoir solution, which was composed of 11% PEG-3350 and 0.25 M KCl. Crystals were mounted after 2 days in 20% PEG-3350, 0.2 M KCl, and 25% glycerol and frozen in liquid nitrogen.

Diffraction datasets were collected at 100 K from the flash-frozen protein crystals using synchrotron radiation at the Diamond Light Source (Didcot, UK) at beamline I24 (*A. dorsata* CEP192^946–1284^) and BM14 (human CEP192^2256–2402^) to resolutions of 3.5 and 1.0 Å, integrated using XDS (*66*) and iMOSFLM (*72*), respectively, and were scaled using AIMLESS (*67*). The *A. dorsata* CEP192^946–1284^ structure was solved by molecular replacement using the *A. dorsata* CEP192^946–1284^ structure prediction from RoseTTAFold (*73*), and the human CEP192^2256–2402^ structure was solved by MAD from a two-wavelength SeMet dataset using the CRANK pipeline (*74*) followed by building of an initial model with BUCCANEER (*75*). Models were constructed by cycles of refinement in PHENIX.REFINE (*76*) and REFMAC (*70*) and manual building in COOT (*69*). Refinement statistics of the final models are summarized in Table 1.

### NMR spectroscopy

#### Drosophila *ASH1 NMR data collection*

All NMR data were collected on a Bruker Avance II 500 MHz triple resonance, pulse field gradient system equipped with a cryoprobe or Bruker Avance II 600 MHz triple resonance, pulse field gradient system. Data were processed using NMRPipe (*77*) and peak picked with Sparky (*78*).

#### Spectroscopy assignment and structure determination

All experiments were performed on ^13^C,^15^N-labeled protein in phosphate-buffered saline (pH 7.0), 1 μM ZnCl_2_, and 1 mM DTT at 20°C. NMR experiments for assignment include ^15^N HSQC, ^13^C HSQC, CBCANH/CBCA(CO)NH, HNCO, HNHA, HCCH-TOCSY, HCCCONH, and CCCONH. All data were peak picked and assigned using Sparky (*78*). Nuclear Overhauser effect (NOE) cross-peaks were observed through ^13^C HSQC-NOESY (aliphatic and aromatic), ^15^N HSQC-NOESY, and ^13^C,^15^N-filtered NOESY. Angular restraints were produced using TALOS+ (*79*), and those consistent with the HNHA data were used in CYANA 2.1 with a combination of manual and autoassigned NOEs.

### In vitro Plk1 kinase reaction

Purified Spd-2^697–1146^ protein (50 μM) and 2 μM Plk1 kinase were added to kinase reaction buffer in a reaction volume of 200 μl. The reaction was then left at 30°C for 1 hour. The kinase reaction buffer contains 50 mM tris (pH 8.0), 150 mM NaCl, 200 μM adenosine 5′-triphosphate, 10 mM MgCl_2_, and 1 mM DTT.

### SEC-MALS analysis

The protein sample (100 μl) was injected onto a Superose 12 Increase 10/300 GL column (GE Healthcare) that was pre-equilibrated by a column buffer composed of 50 mM tris (pH 8.0), 150 mM NaCl, and 1 mM DTT. The measurement was performed at indicated protein concentrations. Light scattering and refractive index were measured using a Dawn Heleos-II light scattering detector and an Optilab-TrEX refractive index monitor, respectively. Analysis was carried out using ASTRA 6.1.1.17 software assuming a *dn*/*dc* value of 0.186 ml/g.

### Circular dichroism

Samples were dialyzed into 10 mM Na*_x_*H*_x_*PO_4_ (pH 8.0) and 0.5 mM TCEP. Buffer-subtracted, averaged spectra (four accumulations) were taken for each sample at 20°C using a Jasco J-815 instrument. Spectra were collected at a protein concentration of 0.2 mg/ml.

### Pfam family building

Pfam families were built from the sequences of Cep192 homologs with domain boundaries defined on the basis of the AF2 (*43*) prediction models. An iterative search was performed using the HMMER package (*80*) with variable thresholds. The newly developed domain families were deposited in Pfam with accession numbers PF22060, PF22064, PF22067, PF22073, PF22074, PF22076, PF22065, and PF22066 and are accessible from the Pfam website (*44*) at InterPro (*81*).

### Phylogenetic analysis

Spd-2 homologs were identified using National Center for Biotechnology Information BLAST and the full Spd-2 sequences of *H. sapiens* and *D. melanogaster* (our null hypothesis being that Spd-2 homologs in other species need not have all ASH domains present). The resulting sequences were then aligned using Jalview version 2.11.3.0 (*82*, *83*) and Clustal Omega (*84*) (to identify all the sequences corresponding to each of the human ASH domains, where they existed). An alignment of all the ASH domain sequences in all the different species was created on MEGA11 using Clustal W with default options, and the evolutionary history was inferred by using the maximum likelihood method and Jones-Taylor-Thornton matrix–based model with Neighbor-Join and BioNJ algorithms and default settings (*85*, *86*). This analysis involved 139 amino acid sequences. There were a total of 267 positions in the final dataset. The final version of the tree was visualized using the free access iTOL online (version 6.8.1) (*87*).
