# Supplementary material for: The conserved Spd-2/CEP192 domain adopts a unique protein fold to promote centrosome scaffold assembly
Source: Sci Adv. 2025 Mar 19;11(12):eadr5744. doi: 10.1126/sciadv.adr5744 (PMC11922060; doi:10.1126/sciadv.adr5744)
Supplement: Supplementary file 1 — Figs. S1 to S7 Legends for movies S1 and S2 [file sciadv.adr5744_sm.pdf]

Science Advances
AAAS

Supplementary Materials for

The conserved Spd-2/CEP192 domain adopts a unique protein fold to promote centrosome scaffold assembly

Liuyi Hu *et al.*

Corresponding author: Zhe Feng, zhefeng@fudan.edu.cn; Mark van Breugel, m.vanbreugel@qmul.ac.uk; Susan M. Lea, susan.lea@nih.gov; Jordan W. Raff, jordan.raff@path.ox.ac.uk

Sci. Adv. **11**, eadr5744 (2025)
DOI: 10.1126/sciadv.adr5744

The PDF file includes:

Figs. S1 to S7
Legends for movies S1 and S2

Other Supplementary Material for this manuscript includes the following:

Movies S1 and S2

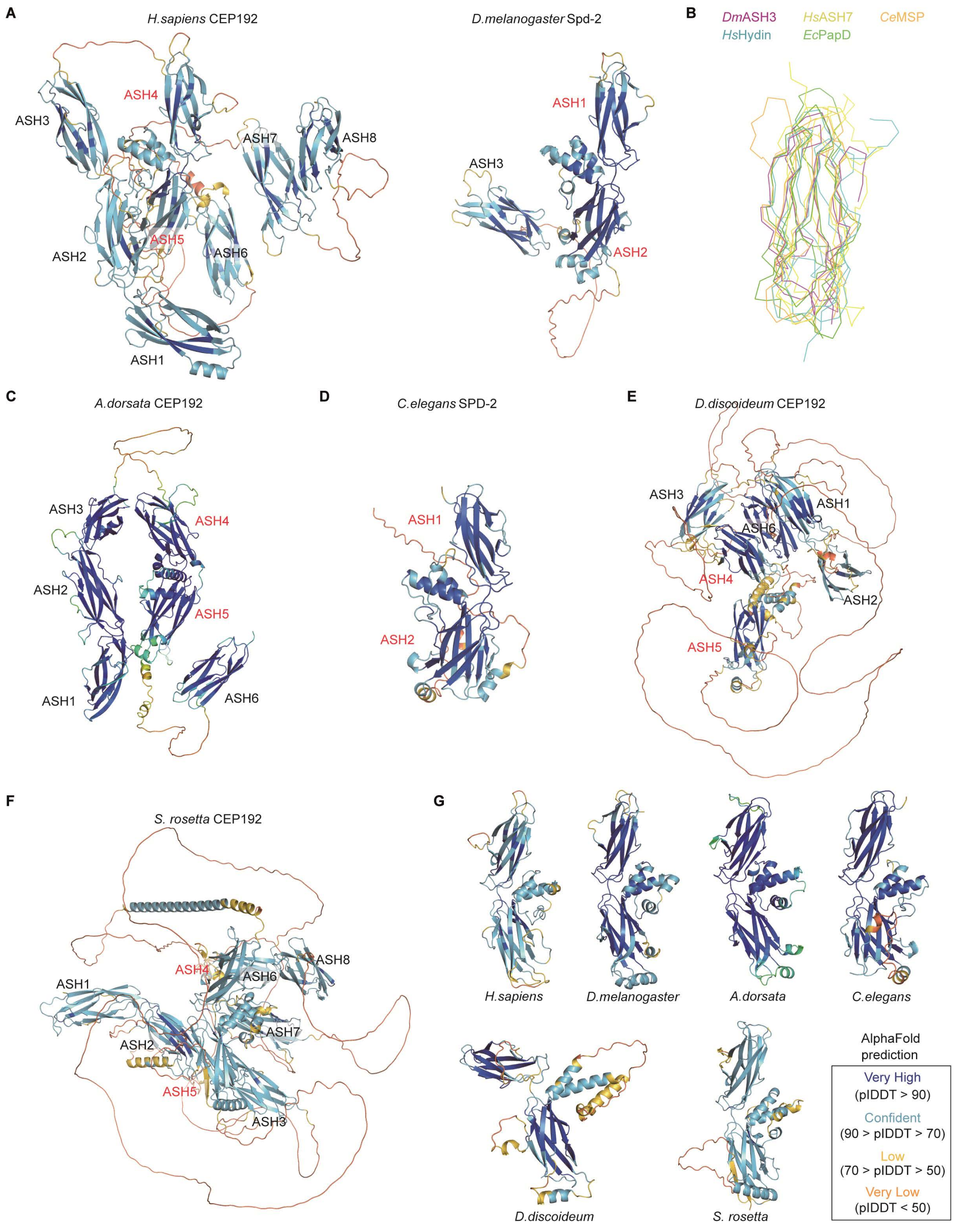

<u>Figure S1.</u>

The structure of individual ASH domains from CEP192/Spd-2 homologs.

(A) Ribbon representation of the structures of individual ASH domains in *Hs*CEP192 and *Dm*Spd-2 as predicted by AF2. **(B)** Structural alignment of the

crystal structures of *Dm*ASH3 (*magenta*), *Hs*ASH7 (*yellow*), the PapD-like domain from human Hydin protein (*cyan*; PDB 2E6J), the PapD protein in *Escherichia coli* (*green*; PDB 3DPA), and the major sperm protein (MSP) in *C. elegans* (*orange*; PDB 1GRW) based on their secondary structures. **(C-F)** Ribbon representation of the structures of the ASH domains from *Ad*Spd-2 (six ASH domains), *Ce*SPD-2 (two ASH domains), *Dd*CEP192 (six ASH domains) and *Sr*CEP192 (eight ASH domains) as predicted by AF2. **(G)** AF2 predicted structures of the SP2D domains from representative CEP192/Spd-2 homologs. All AF2 predictions are colour-coded by the pLDDT values indicating the confidence level of the prediction. ASH domains that constitute the evolutionarily conserved SP2D domain are highlighted in *red*.

A

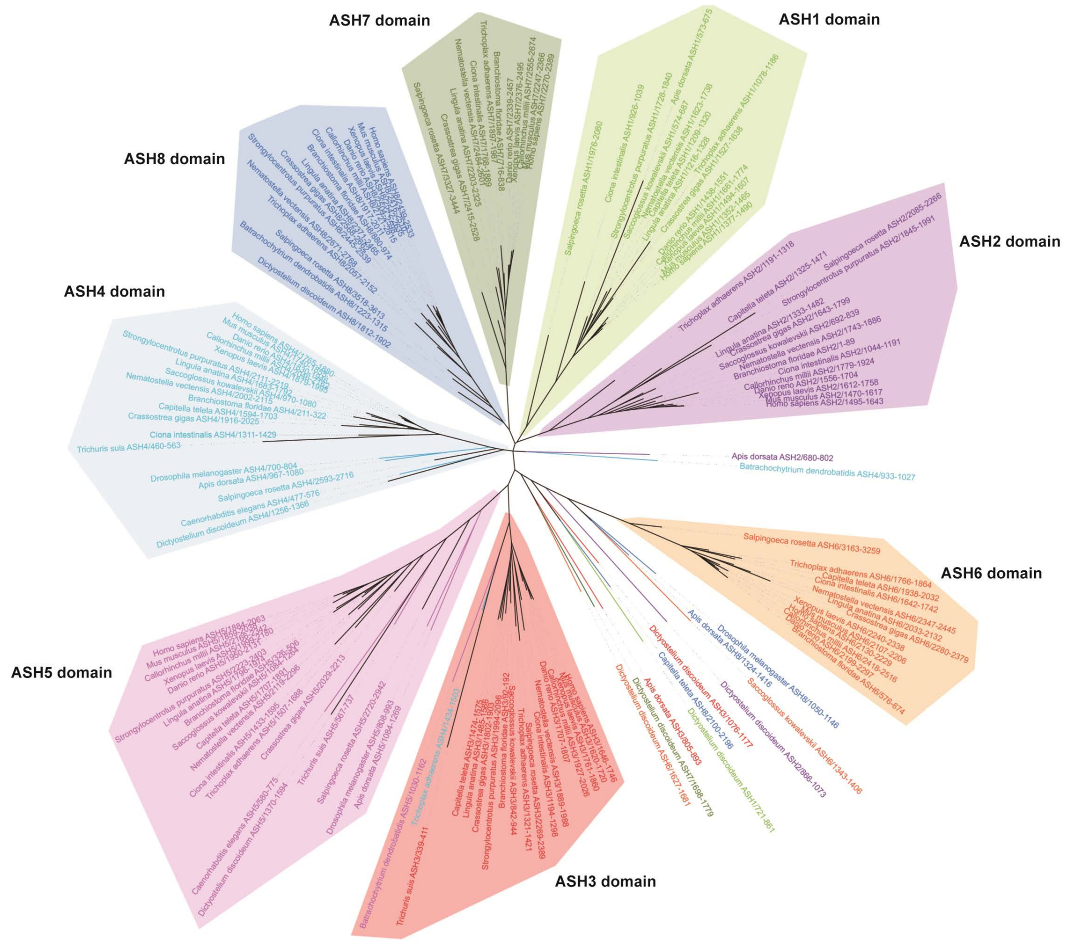

B

		Species	ASH domains							
Metazoa	Chordata	Homo sapiens	1	2	3	4	5	6	7	8
		Mus musculus								
		Xenopus laevis								
		Danio rerio								
		Callorhinchus milii								
		Branchiostoma floridae	X							
		Ciona intestinalis								
		Saccoglossus kowalevskii							X	X
		Strongylocentrotus purpuratus						X	X	
	Ecdysozoa	Apis dorsata		Unclear	Unclear			X	X	ASH6-like
		Drosophila melanogaster	X	X	X			X	X	ASH6-like
		Trichuris suis	X	X				X	X	X
		Caenorhabditis elegans	X	X	X			X	X	X
		Capitella teleta							X	Unclear
		Crassostrea gigas								
		Lingula anatina								
		Nematostella vectensis								
		Trichoplax adhaerens				ASH3-like				
		Salpingoeca rosetta								
		Batrachochytrium dendrobatidis	X	X	X	Unclear	ASH3-like	X	X	
		Dictyostelium discoideum	Unclear	Unclear	Unclear			Unclear	Unclear	

Figure S2.

Phylogenetic analysis of ASH domains from CEP192/Spd-2 homologs. (A)

Phylogenetic tree of ASH domain amino acid sequences from a variety of

species. Search with the Pfam profile HMMs allowed identification of these domains across different phyla, revealing their conservation and distribution in different species. Most ASH sequences clade according to domain types, indicating that ASH domain duplication preceded the divergence of choanoflagellates (like *S. rosetta*) from metazoans. During evolution, individual ASH domains appear to have diverged, been lost, and/or regained via duplication, leading to sequences appearing on the phylogenetic tree outside of their expected domain clade. Branches of diverging ASH sequences are highlighted in the color of their expected domain type. **(B)** Summary table of changes in ASH domain number and identity during evolution. The domain numbering here is based on their structural (i.e. ASH4 and ASH5, the first and second ASH domains of SP2D) and/or positional similarity to the ASH domains in human. Of particular note, we observe a loss of most ASH domains in Ecdysozoa (highlighted by *yellow box*). The ASH domains that have been maintained (mainly ASH4 and ASH5) diverge significantly from homologues in other species. When they appear in their domain clade more basally than expected, this is indicated with their corresponding color being in a lighter tone. *S. rosetta* presents all eight ASH domains, whereas *D. discoideum* potentially presents ASH4, ASH5, ASH8, and five other ASH domains that do not clearly correlate with their expected homologues.

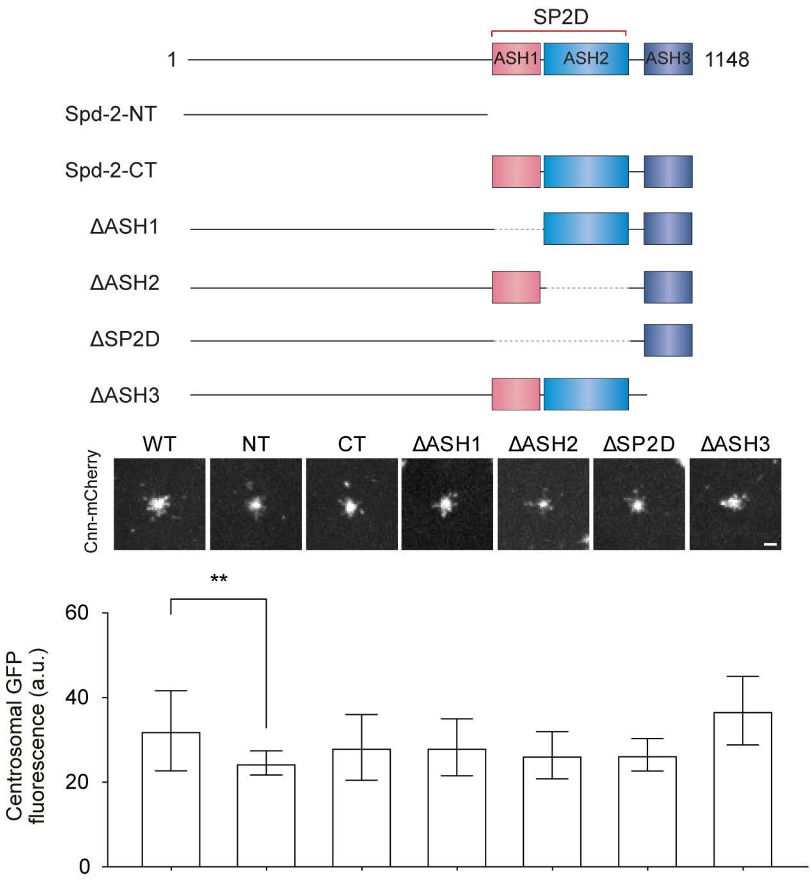

Figure S3.

Deletion of the SP2D domain mildly perturbs Cnn scaffold assembly even in the presence of WT Spd-2. Graphs quantify the centrosomal fluorescence intensity of RFP-Cnn in embryos expressing WT or various truncation or deleted forms of Spd-2-GFP, illustrated in the schematics above the graph. As described in the main text, all of these mutant forms of Spd-2 (apart from ΔASH3) localize very poorly to centrosomes so, even though they are unable to form a Spd-2 scaffold efficiently, we would only expect them to perturb the centrosomal assembly of the Cnn scaffold very mildly in these embryos, which express endogenous WT Spd-2, as we observe.

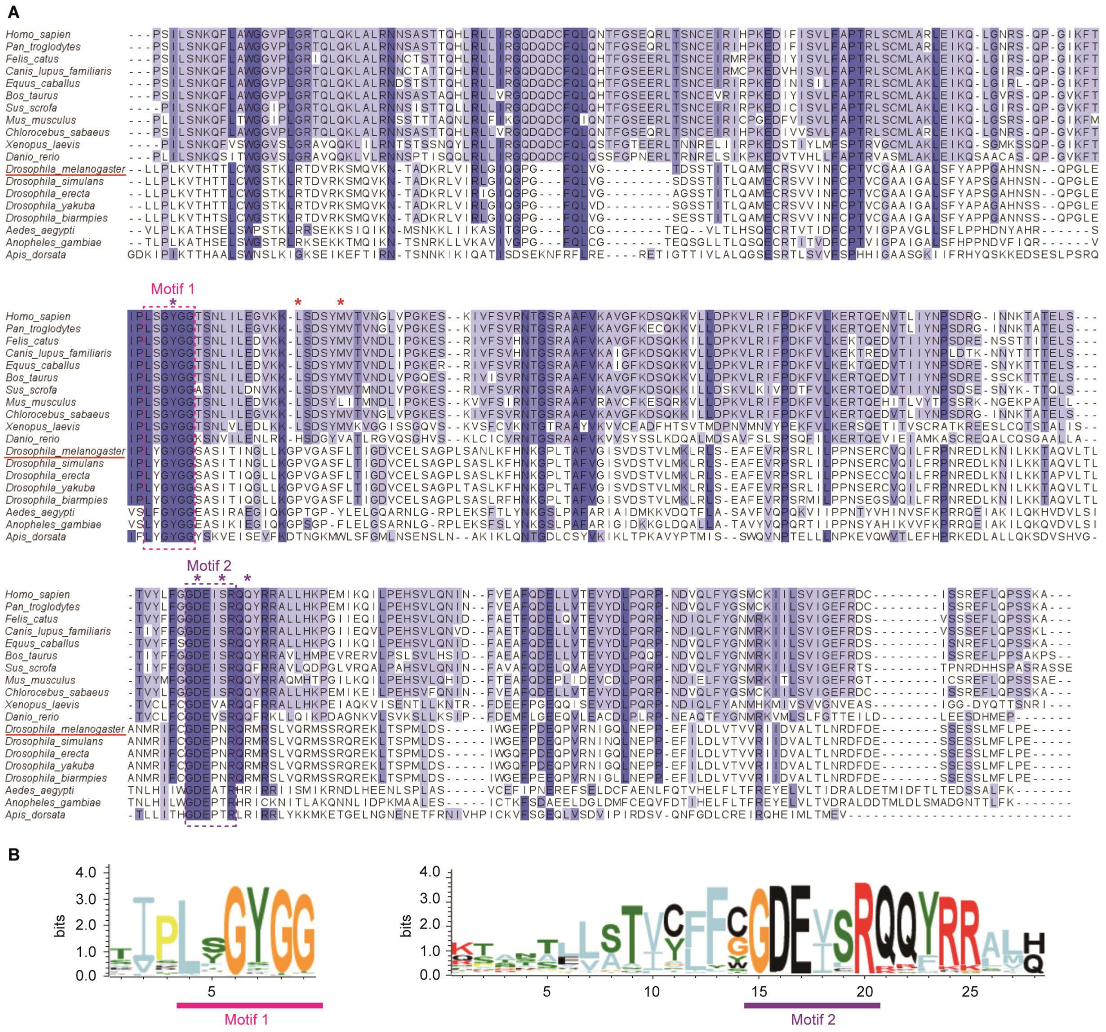

Figure S4.

A multiple sequence alignment (MSA) of the SP2D domain. (A) Multiple sequence alignment of the SP2D domain. Boxes indicate the conserved Motif 1 (*pink*) and Motif 2 (*purple*). Residues highlighted by *purple* asterisks (*) indicate residues that were substituted with Alanine (see main text and Figure 7). The P817 and F822 residues that were mutated to Glu in *Dm*SPD (analysed in Figure 9) are highlighted with a *red* asterisk. **(B)** Sequence logos of Motif1 and Motif2, representing the sequence conservation found in the SP2D. Sequence logos were generated from the Pfam SEED alignments of the

families PF22073 and PF22074 that represent ASH4 and ASH5 of human CEP192.

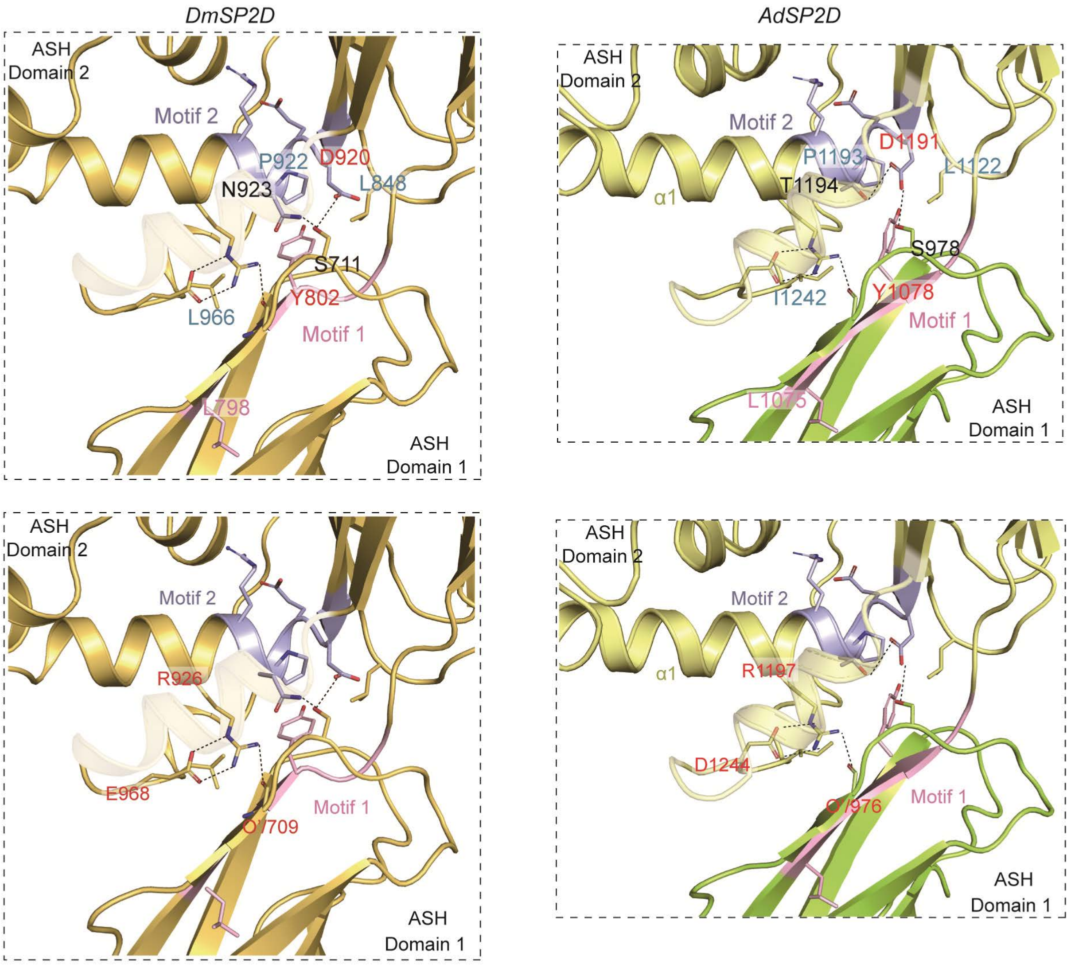

<u>Figure S5.</u>

The interactions within the AF2-predicted *Dm*SP2D are similar to those observed in the *Ad*SP2D crystal structure. Panels show ribbon and stick representations of the *Dm*SP2D (left panels, AF2 prediction) and *Ad*SP2D (right panels, crystal structure), highlighting interactions that maintain the extended cradle structure. The packing interactions in the fly structure are largely similar to those observed in the *Apis* structure. The first major contact is centred

around the conserved Y802 and D920 within Motif 1 (*pink*) and Motif 2 (*purple*), respectively, and the structure is further stabilised by N923 making a hydrogen bond with S711 on the first ASH domain, and by electrostatic interactions between the side chain of R926 with the side chain of E968 and the backbone-carbonyl oxygen of Trp709.

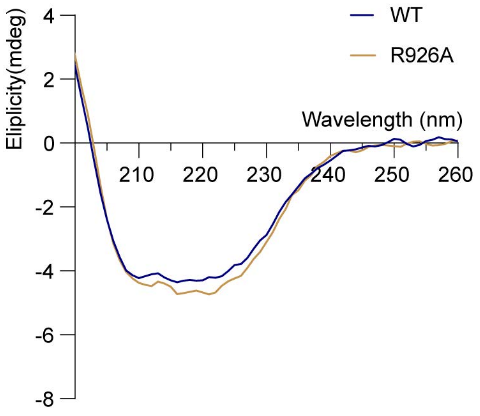

Figure S6.

Biophysical characterisation of WT and R926A mutant *Dm*SP2D constructs. Circular Dichroism (CD) analysis of recombinant, purified WT or R926A mutant SP2D protein. This analysis indicates that the overall fold of the mutant protein is not strongly disrupted by the R926A mutation.

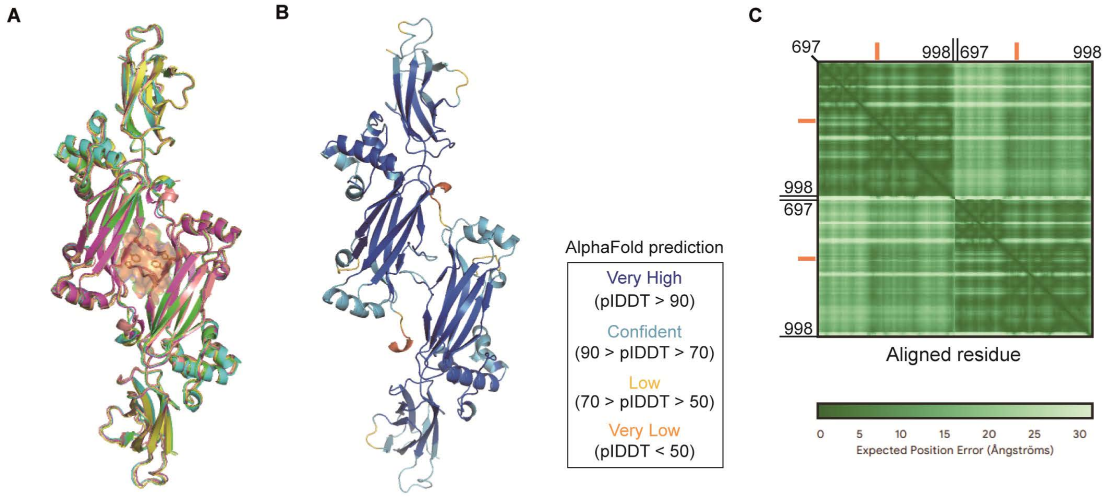

Figure S7.

Summary of results from an AF3 prediction of a putative *Dm*SP2D dimer.

(A) Ribbon diagrams show how the five highest ranking structure-predictions (each shown in a different colour) are near-perfectly aligned into a highly converged configuration. The hydrophobic patch predicted for SP2D dimerisation is highlighted in *orange*. **(B)** AF3 prediction model of the *Dm*SP2D dimer is colour-coded by the pLDDT values indicating the confidence level of the prediction. **(C)** Predicted Aligned Error (PAE) plot for the AF3 predicted dimer structure. PAE is a confidence measure for the relative position of any two residues within the predicted structure. Darker green colours indicate higher confidence levels. The predicted dimerization interface is highlighted with an *orange* bar. Index: amino acid residues.

Supplementary Movie 1.

FRAP analysis for GFP tagged Spd-2 WT or NT truncation mutant. FRAP analysis showing the fluorescence recovery from GFP tagged Spd-2 WT or NT truncation mutant at the centrosome after photobleaching.

Supplementary Movie 2.

FRAP analysis for GFP tagged Spd-2 WT or ΔASH3 deletion mutant. FRAP analysis showing the fluorescence recovery from GFP tagged Spd-2 WT or ΔASH3 deletion mutant at the centrosome after photobleaching.
